# Comparative Heterochromatin Profiling Reveals Conserved and Unique Epigenome Signatures Linked to Adaptation and Development of Malaria Parasites

**DOI:** 10.1016/j.chom.2018.01.008

**Published:** 2018-03-14

**Authors:** Sabine A. Fraschka, Michael Filarsky, Regina Hoo, Igor Niederwieser, Xue Yan Yam, Nicolas M.B. Brancucci, Franziska Mohring, Annals T. Mushunje, Ximei Huang, Peter R. Christensen, Francois Nosten, Zbynek Bozdech, Bruce Russell, Robert W. Moon, Matthias Marti, Peter R. Preiser, Richárd Bártfai, Till S. Voss

**Affiliations:** 1 Department of Molecular Biology, Faculty of Science, Radboud University, 6525 GA Nijmegen, the Netherlands; 2 Department of Medical Parasitology and Infection Biology, Swiss Tropical and Public Health Institute, 4051 Basel, Switzerland; 3 University of Basel, 4001 Basel, Switzerland; 4 School of Biological Sciences, Nanyang Technological University, Singapore 637551, Singapore; 5 Institute of Infection, Immunity and Inflammation, University of Glasgow, Glasgow G12 8QQ, UK; 6 Department of Immunology and Infectious Diseases, Harvard TH Chan School of Public Health, Boston, MA 02155, USA; 7 Department of Immunology and Infection, London School of Hygiene and Tropical Medicine, London WC1E 7HT, UK; 8 Shoklo Malaria Research Unit, Mahidol-Oxford Tropical Medicine Research Unit, Faculty of Tropical Medicine, Mahidol University, Mae Sot 63110, Thailand; 9 Centre for Tropical Medicine and Global Health, Nuffield Department of Medicine Research Building, University of Oxford Old Road Campus, Oxford OX3 7FZ, UK; 10 Department of Microbiology and Immunology, University of Otago, Dunedin 9054, New Zealand

**Keywords:** malaria, *Plasmodium*, heterochromatin, HP1, antigenic variation, gametocytes, host-parasite interaction, epigenetics, gene silencing, sexual differentiation

## Abstract

Heterochromatin-dependent gene silencing is central to the adaptation and survival of *Plasmodium falciparum* malaria parasites, allowing clonally variant gene expression during blood infection in humans. By assessing genome-wide heterochromatin protein 1 (HP1) occupancy, we present a comprehensive analysis of heterochromatin landscapes across different *Plasmodium* species, strains, and life cycle stages. Common targets of epigenetic silencing include fast-evolving multi-gene families encoding surface antigens and a small set of conserved HP1-associated genes with regulatory potential. Many *P*. *falciparum* heterochromatic genes are marked in a strain-specific manner, increasing the parasite's adaptive capacity. Whereas heterochromatin is strictly maintained during mitotic proliferation of asexual blood stage parasites, substantial heterochromatin reorganization occurs in differentiating gametocytes and appears crucial for the activation of key gametocyte-specific genes and adaptation of erythrocyte remodeling machinery. Collectively, these findings provide a catalog of heterochromatic genes and reveal conserved and specialized features of epigenetic control across the genus *Plasmodium*.

## Highlights

• Multi-gene families are common targets of heterochromatin in malaria parasites
• Conserved heterochromatic genes are rare and tend to have regulatory function
• Heterochromatin is stable during asexual replication but variable between strains
• Gametocyte differentiation is linked to changes in the heterochromatin landscape

## Graphical Abstract

**Figure:**
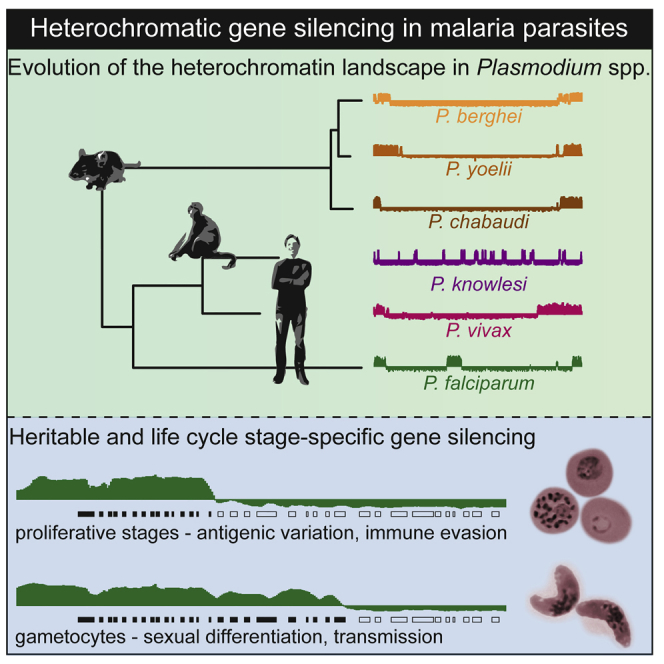

## Teaser

Fraschka, Filarsky et al. performed a genome-wide characterization of heterochromatin organization across multiple species, strains, and life cycle stages of malaria parasites. This revealed that heterochromatic gene silencing is a conserved strategy to drive clonal variation of surface antigens and to control life cycle stage transitions and cell differentiation.

## Introduction

Malaria is caused by unicellular eukaryotes of the genus *Plasmodium* that belongs to an ancient group of obligate endoparasites known as Apicomplexa. The *Plasmodium* genus comprises a few hundred species infecting birds, reptiles, or mammals, and their radiation is estimated to have occurred about 130 million years ago (Perkins, 2014). Members of the *Vinckeia* subgenus parasitize non-primate mammals, among which rodents and bats are the most abundant. This group includes parasites of rodents such as *Plasmodium berghei*, *Plasmodium yoelii*, *Plasmodium Chabaudi*, and *Plasmodium vinckei*, which serve as important models to interrogate *Plasmodium* biology. Parasites belonging to the subgenera *Plasmodium* and *Laverania* infect humans or other primates. Five species are known to naturally infect humans, namely *Plasmodium vivax*, *Plasmodium ovale*, *Plasmodium malariae*, *Plasmodium knowlesi* (all members of the *Plasmodium* clade), and *Plasmodium falciparum* (*Laverania* clade).

Malaria parasites of mammals are transmitted between their intermediate hosts by female *Anopheles* mosquitoes. Their life cycle is complex, involving several stage transitions and replication phases as well as colonization of different cell types and tissues. In the bloodstream, parasites invade red blood cells (RBCs) and undergo intracellular multiplication via schizogony, which involves progression through the ring and trophozoite stages followed by multiple nuclear divisions before a single cytokinesis event leads to the production of up to 32 merozoites ready to invade other RBCs. Repeated rounds of these cycles are responsible for all malaria-related morbidity and mortality. For malaria transmission to occur, mosquitoes must ingest male and female gametocytes with their blood meal. These sexual precursors emerge at a low rate from the proliferating pool of blood stage parasites and are essential to complete sexual reproduction and subsequent sporozoite formation in the mosquito vector. Upon injection into the skin through a mosquito bite, sporozoites migrate to the liver, undergo intra-hepatic schizogony, and release over 10,000 merozoites that commence blood stage infection.

Proteins involved in functions at the host-parasite interface have been key to the evolutionary success of malaria parasites (Swapna and Parkinson, 2017). Genes encoding such factors comprise up to 15% of all parasite genes and belong to various dynamically evolving multi-gene families (Reid, 2015). Characteristic features of these gene families are that they (1) primarily encode proteins exported to the RBC; (2) display high levels of sequence polymorphism between paralogs and across strains, and substantial differences in copy number between species; (3) mostly locate to subtelomeric gene arrays (with the exception of *P*. *knowlesi* where they occur throughout the genome); and (4) are often species- or clade-specific (Pain et al., 2008, Reid, 2015). A prime example of species-specific gene families is the 60-member *var* gene family in *P*. *falciparum*. Each *var* gene encodes a variant of the major surface antigen *P*. *falciparum* erythrocyte membrane protein 1 (PfEMP1) that mediates adhesion of infected RBCs (iRBCs) to several host receptors (Smith et al., 2013). Members of gene families represented in multiple species include the *Plasmodium* interspersed repeat (*pir*) genes (Cunningham et al., 2010), *fam-a*, *-b*, *-c* genes (Otto et al., 2014), *Plasmodium* helical interspersed subtelomeric (*phist*) genes (Sargeant et al., 2006, Warncke et al., 2016), or reticulocyte-binding-like (*rbl*) genes (Gunalan et al., 2013). Independent of their size and species distribution, these gene families provide a fertile ground for genetic diversification and are a driving force of evolutionary adaptation.

A number of studies conducted in *P*. *falciparum* showed that these multi-gene families are located in heterochromatin (Flueck et al., 2009, Lopez-Rubio et al., 2009, Salcedo-Amaya et al., 2009). Heterochromatin is characterized by trimethylation of lysine 9 on histone H3 (H3K9me3) and the consequent binding of heterochromatin protein 1 (HP1), a conserved regulator of heterochromatin formation and heritable silencing (Lomberk et al., 2006). *P*. *falciparum* encodes a single HP1 protein termed PfHP1 (Perez-Toledo et al., 2009, Flueck et al., 2009). In asexual blood stage parasites, PfHP1/H3K9me3 demarcate large heterochromatic domains in all subtelomeric regions and in a few internal regions of some chromosomes (Flueck et al., 2009, Lopez-Rubio et al., 2009, Salcedo-Amaya et al., 2009). These heterochromatic domains are virtually confined to non-syntenic regions and include over 400 genes, almost all of which are members of multi-gene families (Flueck et al., 2009). As a consequence, these genes are subject to clonally variant expression, providing the parasites with a strong potential for phenotypic diversification and rapid adaptation for instance through antigenic variation or expression of alternative invasion ligands or nutrient transporters (Rovira-Graells et al., 2012, Voss et al., 2014). In addition, a few single genes are also associated with PfHP1, some of which have orthologs in other *Plasmodium* species (Flueck et al., 2009). One of these loci encodes the transcription factor AP2-G, the master regulator of gametocytogenesis (Kafsack et al., 2014, Sinha et al., 2014). PfHP1-dependent silencing of *pfap2-g* prevents sexual commitment, while activation of this locus triggers sexual conversion and subsequent gametocyte differentiation, thus facilitating parasite transmission to the mosquito vector (Kafsack et al., 2014, Sinha et al., 2014, Brancucci et al., 2014, Coleman et al., 2014).

These and other studies provided clear evidence that epigenetic regulation, particularly heterochromatin formation, is central to adaptation and survival of malaria parasites. To date, however, heterochromatin organization has almost exclusively been investigated in *P*. *falciparum* strain 3D7 blood stage schizonts. It is currently unknown whether the heterochromatin landscape differs between *P*. *falciparum* strains, whether other *Plasmodium* spp. display similar heterochromatin landscapes, or to what extent HP1 contributes to life cycle stage transitions and parasite differentiation.

## Results

### Conserved and Species-Specific Aspects of the Heterochromatin Landscape across the *Plasmodium* Genus

**Figure 1:**
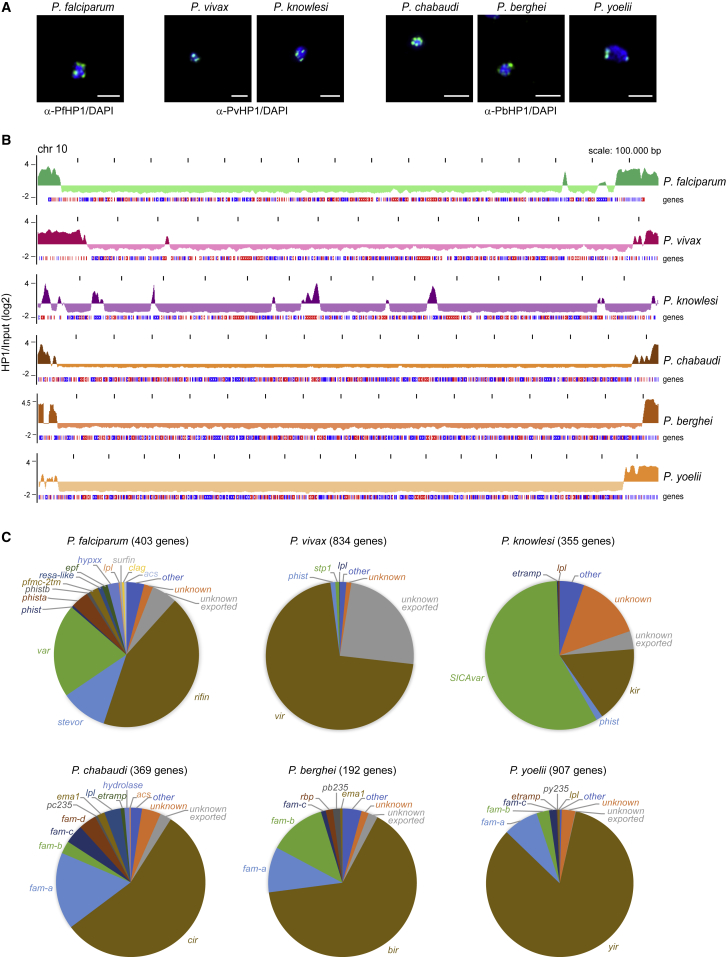
HP1 Localization and Genome-wide HP1 Occupancy in Six Different *Plasmodium* Species (A) IFAs showing HP1 localization (green) in *P*. *falciparum* (α-PfHP1 antibodies), *P*. *vivax*, and *P*. *knowlesi* (α-PvHP1 antibodies), and *P*. *chabaudi*, *P*. *berghei*, and *P*. *yoelii* (α-PbHP1 antibodies) trophozoites. Nuclei were stained with DAPI (blue). Scale bars, 2.5 μm. (B) Log_2_-transformed ChIP/input ratio tracks from schizont stages of six *Plasmodium* species. Coding sequences are shown as blue (sense strand) and red (antisense strand) boxes. (C) Relative composition of heterochromatic genes in six *Plasmodium* species, classified into multi-gene families or groups of “unknown,” “unknown exported,” and “other.” Numbers indicate the total number of high-confidence heterochromatic genes. See also Figures S1–S3; Tables S1 and S2.

To investigate evolutionary aspects of heterochromatin organization, we profiled genome-wide HP1 occupancy in multiple *Plasmodium* species by chromatin immunoprecipitation sequencing (ChIP-seq). For *P*. *falciparum* we used our recently generated polyclonal rabbit α-PfHP1 antibody (Brancucci et al., 2014). Guided by a phylogenetic tree constructed from HP1 orthologs (Figure S1), we generated additional polyclonal rabbit antibodies against PvHP1 (to study HP1 in *P*. *vivax* and *P*. *knowlesi*) and PbHP1 (to study HP1 in *P*. *berghei*, *Plasmodium chabaudi*, and *P*. *yoelii*). Immunofluorescence assays (IFAs) using these antibodies visualized punctate signals in the nuclei of all species that are reminiscent of the perinuclear chromosome end clusters observed in *P*. *falciparum* (Brancucci et al., 2014) (Figures 1A and S1). In western blot analyses these antibodies detected a protein of the expected size of HP1 in each species (for *P*. *vivax* western blot analysis was not performed due to lack of a suitable parasite sample) (Figure S1).

Using these antibodies we mapped HP1 occupancy in schizonts of *P*. *falciparum* strain 3D7, *P*. *berghei* ANKA, *P*. *chabaudi chabaudi* AS, *P*. *yoelii yoelii* YM, and *P*. *knowlesi* clone A1-C.1 (Moon et al., 2013), as well as *P*. *vivax* field isolates. In all species HP1 predominantly localizes to subtelomeric heterochromatic domains on all chromosomes and to a few internal regions on some chromosomes (Figures 1B and S2). The only exception is *P*. *knowlesi*, where subtelomeric occupancy is much less pronounced but numerous chromosome-internal HP1-demarcated domains are observed (Figures 1B and S2). We next calculated HP1 enrichment values for each gene and employed a binomial Gaussian mixture model to call HP1-associated genes with high confidence (Figure 1C and Table S1). HP1 occupancy in *P*. *falciparum* is largely restricted to the *var*, *rif*, *stevor*, *phist*, *pfmc-2tm*, and other gene families encoding known or predicted exported proteins in accord with a previous report (Flueck et al., 2009). In *P*. *vivax* most HP1-occupied genes belong to the *vir* family, and members of the *cir*, *bir*, and *yir* families make up the majority of HP1-associated genes in *P*. *chabaudi*, *P*. *berghei*, and *P*. *yoelii*, respectively. The dispersed HP1-demarcated domains in *P*. *knowlesi* capture the *kir* and *SICAvar* families and the interstitial telomere repeat sequences (ITSs) that are linked to these loci throughout the genome (Pain et al., 2008) (Figures 1C and S3; Table S1). Most other HP1-associated genes in *P*. *vivax*, *P*. *knowlesi*, and the three rodent-infecting species are members of gene families encoding other known or predicted exported proteins including *phist*, *stp1*, *fam-a*, *fam-b*, and *fam-c* genes (Figure 1C and Table S1) (Reid, 2015). Moreover, several species possess small heterochromatic gene families involved in RBC invasion such as the *pc235*, *pb235*, and *py235* genes encoding rhoptry proteins (*P*. *chabaudi*, *P*. *berghei*, *P*. *yoelii*) (Iyer et al., 2007), or in metabolism such as *lpl* genes encoding lysophospholipases (*P*. *falciparum*, *P*. *vivax*, *P*. *knowlesi*, *P*. *chabaudi*, and *P*. *yoelii*) and *acs* genes encoding acyl-coenzyme A synthetases (*P*. *falciparum* and *P*. *chabaudi*).

**Figure 2:**
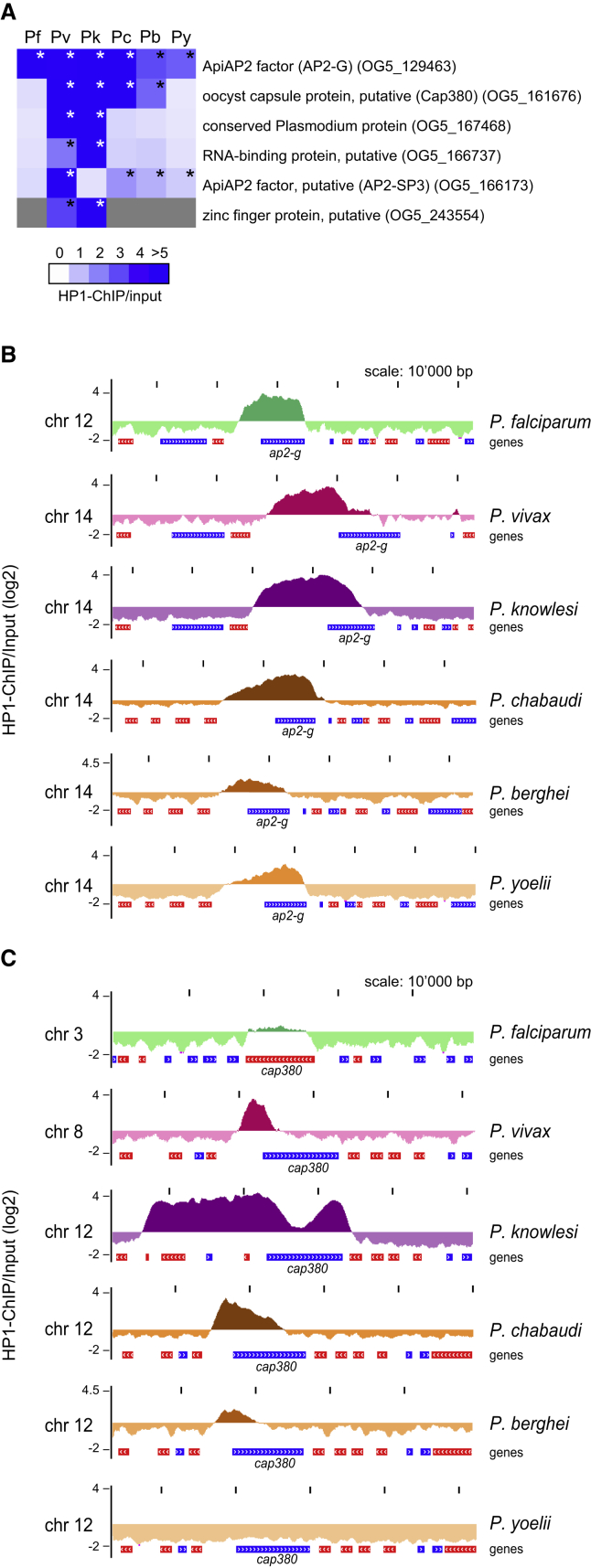
Conserved Single-Copy Genes Associated with HP1 in More Than One Species (A) HP1 enrichment values for conserved syntenic orthologs in six *Plasmodium* species. Asterisks denote high-confidence heterochromatic genes (p > 0.99999). (B) HP1 enrichment tracks over the *ap2-g* locus in six *Plasmodium* species. Coding sequences are shown as blue (sense strand) and red (antisense strand) boxes. (C) HP1 enrichment tracks over the *cap380* locus in six *Plasmodium* species. See also Tables S1 and S2.

All species also contain a few HP1-associated genes encoding proteins involved in the regulation of gene expression, vesicular transport, cell division, RBC invasion, and sexual development or transmission (summarized in the category “other”; Figure 1C and Table S1). Notably, while the multi-gene families have no or limited orthology, most of these genes have orthologs including some with conserved synteny across species. The extent of HP1 enrichment at these loci varied across species and most were bound by HP1 only in one species (Tables S1 and S2). However, six conserved syntenic orthologs were associated with HP1 in more than one species (Figure 2A). Four of them encode putative transcriptional or post-transcriptional regulators of gene expression, namely the ApiAP2 TFs AP2-G (Kafsack et al., 2014, Sinha et al., 2014) and AP2-SP3/AP2-Tel (Modrzynska et al., 2017, Sierra-Miranda et al., 2017), an RNA-binding protein, and a CCCH-type zinc finger (ZnF) protein that is only conserved in *P*. *vivax* and *P*. *knowlesi*. Interestingly, *ap2-g* was the only gene with clear HP1 enrichment in all species (Figure 2B), underscoring its crucial role in controlling the switch to sexual differentiation (Kafsack et al., 2014, Sinha et al., 2014, Brancucci et al., 2014, Coleman et al., 2014). *ap2-sp3/ap2-tel* was bound by HP1 in *P*. *vivax* and the three species infecting rodents. The two genes encoding the ZnF and RNA-binding proteins and a gene encoding a conserved *Plasmodium* protein of unknown function were significantly enriched only in the *P*. *vivax/knowlesi* clade (Figure 2A). *cap380*, encoding an oocyst capsule protein essential for oocyst development in *P*. *berghei* (Srinivasan et al., 2008), is associated with HP1 in *P*. *vivax*, *P*. *knowlesi*, *P*. *berghei*, and *P*. *chabaudi*, and partially marked in *P*. *falciparum*, but not in *P*. *yoelii* (Figure 2C).

In summary, in all *Plasmodium* species examined most HP1-enriched genes belong to species-, clade- or pan-specific multi-gene families with documented or probable functions in antigenic variation, immune evasion, or host cell invasion. In addition, each species contains a small number of HP1-associated single-copy genes, many of which are conserved in other *Plasmodium* spp. and have known or predicted roles in fundamental parasite biology.

### *P*. *knowlesi* Parasites Proliferating in Macaque or Human RBCs Display Altered PkHP1 Occupancy at Several Loci

**Figure 3:**
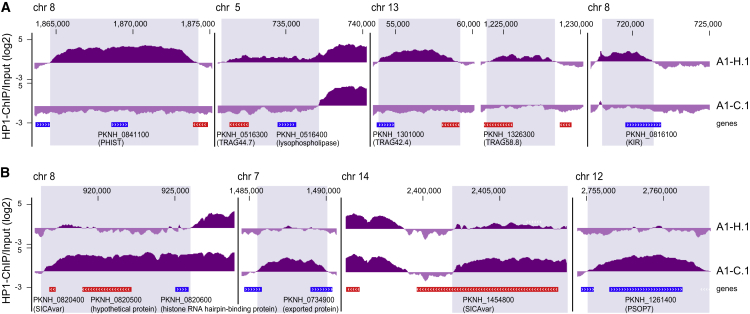
Genes Differentially Marked by PkHP1 in *P*. *knowlesi* Parasites Invading Human or Macaque RBCs (A) HP1 enrichment tracks over the six genes showing >2.5-fold increased HP1 occupancy in *P*. *knowlesi* A1-H.1 compared with A1-C.1. Coding sequences are shown as blue (sense strand) and red (antisense strand) boxes. (B) HP1 enrichment tracks over the six genes showing >2.5-fold increased HP1 occupancy in *P*. *knowlesi* clone A1-C.1 compared with A1-H.1. See also Table S3.

*P*. *knowlesi* parasites have been adapted to continuous *in vitro* culture in human RBCs (Moon et al., 2013, Lim et al., 2013). We reasoned that the adaptation to growth in human RBCs may have involved epigenetic changes. We therefore compared the PkHP1 binding profiles of *P*. *knowlesi* clones A1-C.1 (see above) and A1-H.1, which have been adapted to long-term *in vitro* culture in *Macaca fascicularis* and human RBCs, respectively (Moon et al., 2013). Only 12 genes were differentially marked by PkHP1 between the two clones (≥2.5-fold change in PkHP1 occupancy) (Table S3). The six loci with higher PkHP1 enrichment in human RBC-adapted parasites encode a KIR protein, a lysophospholipase, a PHIST protein, and three tryptophan-rich antigens (TRAGs) (Figure 3A and Table S3). The six genes with reduced PkHP1 occupancy in human RBC-adapted parasites encode two members of the SICAvar family, a protein of unknown function, a predicted exported protein, the secreted ookinete protein PSOP7, and a putative histone RNA hairpin-binding protein (Figure 3B and Table S3). Of note, TRAG proteins interact with RBC receptors and have proposed roles in invasion (Tyagi et al., 2015, Zeeshan et al., 2015), and PHIST proteins play central roles in RBC remodeling (Warncke et al., 2016). Furthermore, the *P*. *knowlesi* gene encoding a *Plasmodium* exported protein of unknown function (PKNH_0734900) has an ortholog in *P*. *vivax*, a parasite that naturally infects humans. Hence, these epigenetic changes may indeed represent signatures of positive selection during adaptation but replicate *in vitro* selection experiments, and further characterization of candidates is needed to test this intriguing possibility experimentally.

### Heterochromatin Organization Is Variable between Different *P*. *falciparum* Strains

**Figure 4:**
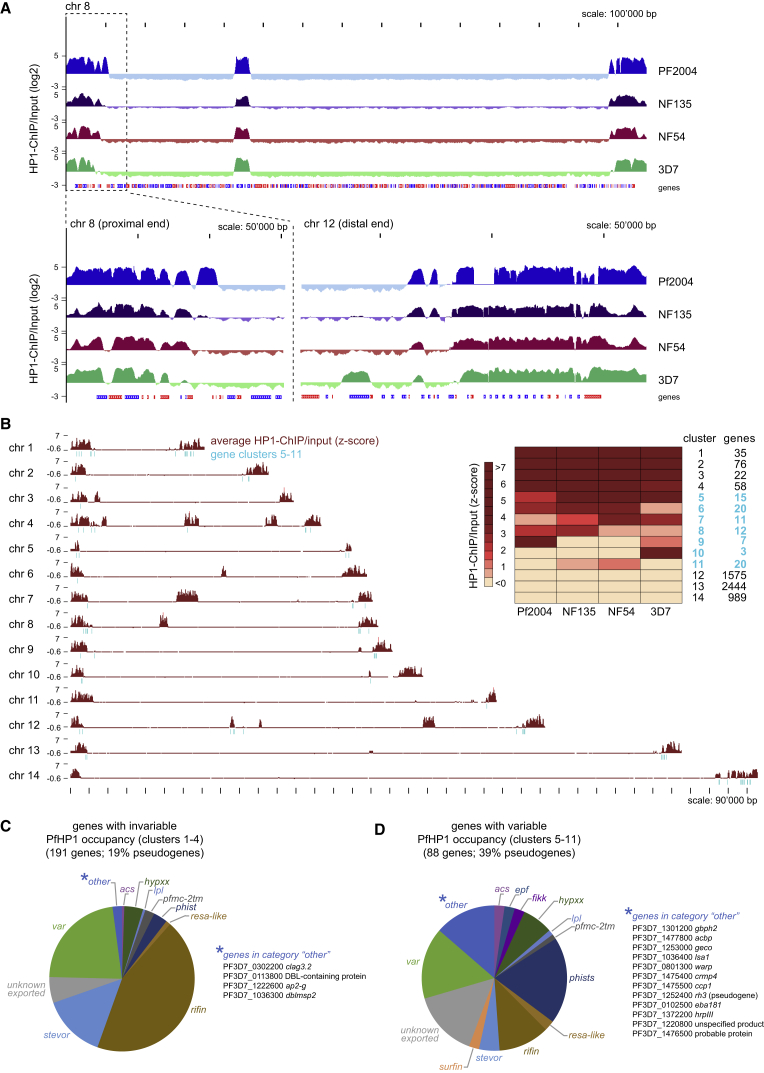
Strain-Specific Differences in Heterochromatin Organization in *P*. *falciparum* Schizonts (A) Log2-transformed ChIP/Input ratio tracks from *P*. *falciparum* strains Pf2004, NF135, NF54 and 3D7 schizonts. Chromosome 8 and zoom-ins of its proximal end and the distal end of chromosome 12 are depicted as representative examples. Coding sequences are shown as blue (sense strand) and red (antisense strand) boxes. (B) Heatmap based on k-means clustering of *Z*-score-transformed ChIP/input ratios calculated for each gene. Clusters containing genes with variable HP1 occupancy are marked in turquoise. Chromosome maps depict the position of variably marked genes (turquoise) in relation to HP1-demaracted heterochromatin (brown tracks; average *Z*-score-transformed ChIP/input ratios across the strains calculated in 1,000-bp windows). (C) Relative composition of invariably marked heterochromatic genes (clusters 1–4), classified into multi-gene families or groups of “unknown exported” and “other.” (D) Relative composition of variably marked heterochromatic genes (clusters 5–11), classified into multi-gene families and groups of “unknown exported” and “other.” See also Figure S4 and Table S4.

We next profiled HP1 occupancy in *P*. *falciparum* schizont stages of strain NF54 (Delemarre and van der Kaay, 1979), the NF54-derived clone 3D7 (Walliker et al., 1987), and the recently culture-adapted Ghanaian strain Pf2004 (Elliott et al., 2007, Brancucci et al., 2015) and Cambodian strain NF135 (Teirlinck et al., 2013). To allow direct comparison of PfHP1 occupancy, we mapped all ChIP-seq reads against the 3D7 genome (PlasmoDB v26). The four strains displayed largely similar heterochromatin organization but distinct PfHP1 occupancy was still evident, predominantly at the border of heterochromatic domains (Figure 4A). Importantly, mapping the Pf2004 ChIP-seq reads against the matching Pf2004 genome revealed that many changes in PfHP1 occupancy occurred in syntenic regions (Figure S4), demonstrating that differences at heterochromatin borders are not solely due to genetic rearrangements.

For identification of genes with altered PfHP1 occupancy, ChIP/input enrichment values were *Z-*score transformed and grouped using k-means clustering (Figure 4B and Table S4). Of all heterochromatic genes (clusters 1–11; 279 genes), one-third displayed variable PfHP1 occupancy across strains (clusters 5–11; 88 genes). Interestingly, most of these genes localize close to heterochromatin boundaries (Figure 4B) and show variation in expression between laboratory lines (Rovira-Graells et al., 2012) (Table S4). While most *var*, *stevor*, and *rifin* genes were stably marked by PfHP1, members of other gene families such as *phist*, *fikk*, or *surfin* and genes encoding unknown exported proteins were over-represented among the variably marked genes (Figures 4C and 4D; Table S4). Pseudogenes were also more abundant in this class, suggesting that they may provide “buffer zones” for heterochromatin reorganization. Variable PfHP1 occupancy was also observed for most PfHP1-associated single-copy genes and small gene families (category “other”) (Figure 4D). This set includes genes encoding proteins implicated in erythrocyte invasion (*eba-181*) (Gilberger et al., 2003), RBC remodeling in gametocytes (*geco*) (Morahan et al., 2011), mosquito midgut invasion (*warp*) (Yuda et al., 2001), sporozoite maturation or egress (*ccp1*, *crmp4*) (Simon et al., 2009, Douradinha et al., 2011), or liver stage development (*lsa1*) (Mikolajczak et al., 2011). Notably, however, four such genes (*ap2-g*, *clag3*.*2*, *dblmsp2*, and another gene encoding a DBL-domain-containing protein) showed stable PfHP1 enrichment in all strains (Figure 4C), suggesting that stable heterochromatin inheritance at these loci provides a selective growth advantage *in vitro*. Indeed, depletion of HP1 from the *pfap2-g* locus leads to cell cycle exit and sexual differentiation (Brancucci et al., 2014). *dblmsp2* encodes a putative invasion factor expressed only in a small fraction of schizonts (Amambua-Ngwa et al., 2012). *clag3*.*2* and its paralog *clag3*.*1* encode related variants of the surface transport channel PSAC (Nguitragool et al., 2011); parasites express either one of the two variants and some preferentially express *clag3*.*1 in vitro* (Cortes et al., 2007, Comeaux et al., 2011). Collectively, these observations highlight a high degree of variability in heterochromatin organization that likely contributes to phenotypic variation of malaria parasites. Interestingly, by analyzing gene expression data from field isolates (Mok et al., 2015) we found that variably marked heterochromatic genes display a significantly higher degree of expression variation compared with euchromatic genes and, to a lesser extent, also to invariably marked genes, suggesting that this relation may be relevant *in vivo* (Figure S4 and Table S4).

### Heterochromatin Organization Is Invariable between Different Stages of Asexual Intra-erythrocytic Development

**Figure 5:**
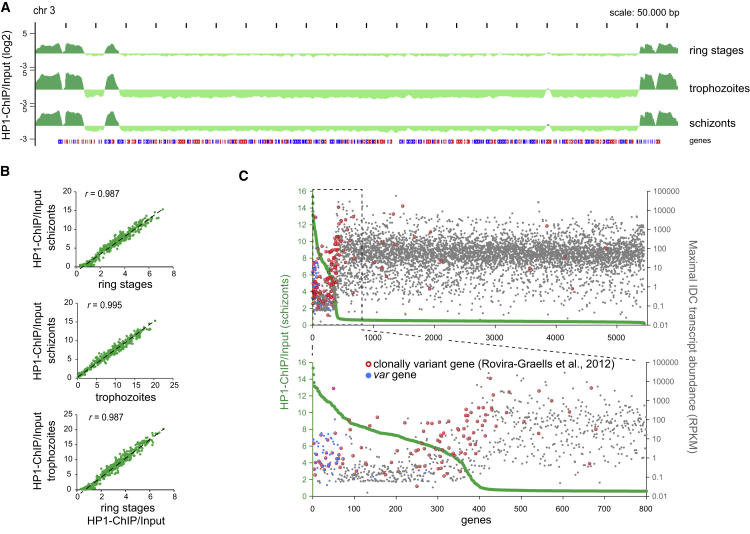
Genome-wide PfHP1 Localization Is Invariable across the IDC (A) Log_2_-transformed ChIP/Input ratio tracks from *P*. *falciparum* 3D7 ring, trophozoite, and schizont stages. Chromosome 3 is depicted as a representative example. Coding sequences are shown as blue (sense strand) and red (antisense strand) boxes. (B) Pairwise comparisons of PfHP1 coverage of individual genes between the three IDC stages. *r*, Pearson correlation values. (C) Scatterplots displaying for each gene the maximum transcript level during the IDC (Kensche et al., 2016) (gray dots) in relation to HP1 occupancy in schizonts (green dots). Genes were sorted according to HP1 occupancy. Genes with clonally variant expression (Rovira-Graells et al., 2012) are marked with a red circle. *var* genes are indicated as blue dots. See also Table S5.

To assess whether and to what extent PfHP1-dependent gene expression contributes to the regulation of gene expression during the intra-erythrocytic developmental cycle (IDC), we mapped PfHP1 occupancy in *P*. *falciparum* 3D7 ring stages, trophozoites, and schizonts. The profiles were highly similar in all three stages (Figure 5A). PfHP1 enrichment values of individual genes were highly correlated and we did not identify any genes with significantly altered PfHP1 occupancy across the IDC (Figure 5B and Table S5). Comparison of our data with an RNA sequencing (RNA-seq) dataset (Kensche et al., 2016) confirmed that most PfHP1-associated genes are expressed at low levels during the IDC (Flueck et al., 2009) and that most clonally variant genes are PfHP1 target genes (Rovira-Graells et al., 2012) (Figure 5C). Interestingly, genes with lower PfHP1 occupancy showed somewhat higher expression, and this set includes many experimentally confirmed clonally variant genes (Rovira-Graells et al., 2012). *var* genes appear to be special in this regard since they show moderate expression despite high PfHP1 occupancy levels. In summary, these results reveal that PfHP1-mediated silencing does not contribute in any major way to the temporal regulation of gene expression during the IDC.

### The Switch from Asexual Proliferation to Sexual Differentiation in *P*. *falciparum* Is Accompanied by Marked Changes in the Heterochromatin Landscape

**Figure 6:**
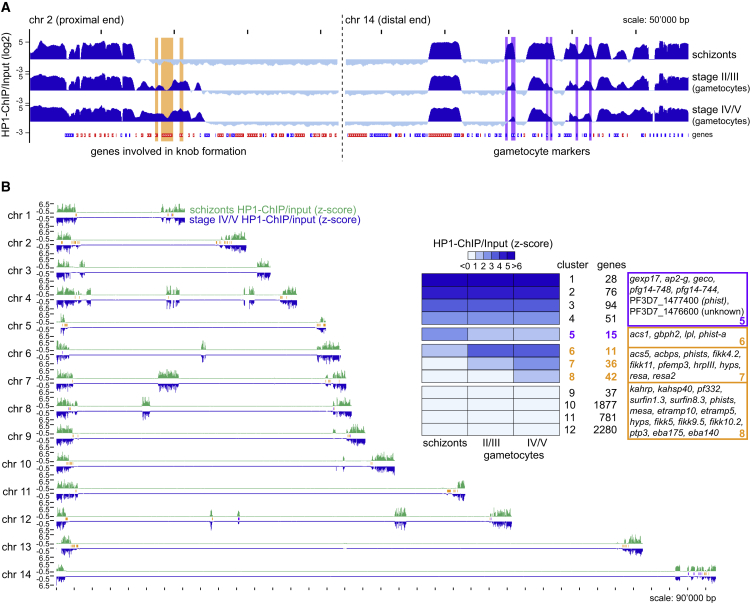
Differences in Heterochromatin Organization between Asexual and Sexual *P*. *falciparum* Blood Stage Parasites (A) Log_2_-transformed ChIP-seq ChIP/input ratio tracks from *P*. *falciparum* Pf2004 schizonts and stage II/III and stage IV/V gametocytes. The proximal end of chromosome 2 is depicted as an example for an expanded heterochromatic domain in gametocytes. Genes involved in knob formation (*kahsp40*, PF3D7_0201800; *pfemp3*, PF3D7_0201900; *kahrp*, PF3D7_0202000) are marked in orange. Early gametocyte markers PF3D7_1476500, PF3D7_1476600, PF3D7_1477300 (*pfg14_744/phist*), PF3D7_1477400 (*phist*), PF3D7_1477700 (*pfg14_748/phista*), and PF3D7_1478000 (*gexp17*) at the distal end of chromosome 14 have reduced PfHP1 occupancy in gametocytes and are marked in purple. (B) Heatmap based on k-means clustering of *Z*-score-transformed ChIP/input ratios calculated for each gene. Examples of genes with reduced (cluster 5) or increased (clusters 6–8) HP1 occupancy in gametocytes are highlighted in purple and orange, respectively. Chromosome maps depict the position of genes with reduced (purple) or increased (orange) HP1 occupancy in gametocytes in relation to PfHP1-demarcated heterochromatin (green tracks and blue inverted tracks are *Z*-score-transformed ChIP/input ratios in schizonts and stage IV/V gametocytes, respectively, calculated in 1,000-bp windows). See also Figure S5 and Table S6.

We performed PfHP1 ChIP-seq experiments on Pf2004 schizonts, stage II/III gametocytes, and stage IV/V gametocytes. Mapping the PfHP1 ChIP-seq reads against both the 3D7 and Pf2004 reference genomes highlighted clear differences in PfHP1 occupancy between asexual and sexual stages that were particularly evident from the expansion of subtelomeric heterochromatic domains in gametocytes (Figures 6A, 6B, and S5).

Calculation of PfHP1 enrichment values followed by *Z-*score transformation and k-means clustering identified 104 genes with altered PfHP1 occupancy between schizonts and gametocytes (clusters 5–8) (Figure 6B and Table S6). Of these, only 15 genes showed reduced PfHP1 occupancy in gametocytes (cluster 5). This set includes *pfap2-g* (Kafsack et al., 2014), the gametocyte-specific gene *pfgeco* (Morahan et al., 2011), and seven additional genes at the distal end of chromosome 14 that include five known markers of early gametocytogenesis (*pfg14_744*, *pfg14_748*, PF3D7_1476600, PF3D7_1477400, *gexp17*) (Eksi et al., 2005, Eksi et al., 2012, Silvestrini et al., 2010) (Figures 6A, 6B, and S5; Table S6).

Clusters 6–8 contain 89 genes specifically bound by PfHP1 in gametocytes (Figures 6A, 6B, and S6; Table S6). Intriguingly, this set is enriched for genes encoding proteins implicated in RBC remodeling. Of particular interest is the subtelomeric region at the left arm of chromosome 2 where the heterochromatic domain is extended by almost 50 kb in gametocytes. This differentially marked region includes three genes encoding proteins involved in knob formation, namely the knob-associated heat-shock protein 40 (KAHsp40) (Acharya et al., 2012), PfEMP3 (Pasloske et al., 1993), and the knob-associated histidine-rich protein (KAHRP) (Pologe and Ravetch, 1986). In addition, five members of the *fikk* family, which encode exported serine-threonine protein kinases implicated in host cell remodeling (Nunes et al., 2007, Kats et al., 2014), are enriched in PfHP1 in gametocytes. Increased PfHP1 occupancy is also observed at the gene encoding MESA, an exported protein of unknown function that binds to the RBC membrane skeleton protein 4.1 (Waller et al., 2003) and at 15 *phist* genes. In summary, these data suggest that heterochromatin remodeling contributes in a major way to the establishment of a gametocyte-specific transcriptional program. It should be noted, however, that the majority of genes differentially expressed between asexual and sexual blood stages are not marked by HP1 in either stage (Young et al., 2005, Flueck et al., 2009), suggesting that sequence-specific transcription factors such as AP2-G and AP2-G2 (Kafsack et al., 2014, Sinha et al., 2014, Yuda et al., 2015) are the main drivers of stage-specific gene expression during sexual differentiation.

## Discussion

We show that heterochromatin formation at chromosome ends and their perinuclear clustering is a conserved feature of chromatin organization across the *Plasmodium* genus. Furthermore, in all six species examined heterochromatin primarily embeds members of the various species-, clade-, or pan-specific multi-gene families with known or predicted roles in antigenic variation and other host-parasite interrelations, independent of chromosomal location. While in *P*. *berghei*, *P*. *chabaudi*, *P*. *yoelii*, and *P*. *vivax* heterochromatin is mostly confined to chromosome ends, *P*. *falciparum* features some additional intra-chromosomal heterochromatic islands, and in *P*. *knowlesi* chromosome-internal HP1-demaracted domains are scattered throughout the genome. These differences in heterochromatin distribution mirror the differences in the genome-wide localization of gene families between the species. These observations, in particular the intriguing association of PkHP1 with the numerous individual *kir* and *SICAvar* loci (Figure S3), lends support to the idea that unknown DNA elements linked to sequences of gene family members may be directly involved in the formation and/or local containment of heterochromatin. The ITS elements found at *kir* and *SICAvar* loci are interesting first candidates to be tested for such putative functions.

Some conserved single-copy genes are subject to HP1-dependent gene silencing in at least one of the species analyzed. Heterochromatinization of these genes may be used to prevent their expression during certain phases of the life cycle and/or to express them in a clonally variant manner to facilitate alternative phenotypes conducive to parasite adaptation. Even though some of these genes display only subtle HP1 enrichment and may represent false-positive hits, this list constitutes a valuable resource for the exploration of genus-, clade-, or species-specific heterochromatic genes with functions in key biological processes (Table S2). Here, we focused our attention on the six syntenic orthologs that are heterochromatic in more than one species. Remarkably, next to *ap2-g* this set includes three other genes with likely roles in regulating gene expression. Of these, AP2-SP3/AP2-Tel has recently been studied in *P*. *falciparum* and *P*. *berghei*. In *P*. *falciparum*, this factor binds the telomeric tract (Sierra-Miranda et al., 2017), and disruption of *ap2-sp3*/*ap2-tel* impairs parasite proliferation (Balu et al., 2010). In *P*. *berghei*, however, AP2-SP3/AP2-Tel is dispensable for intra-erythrocytic growth and sexual development but essential for sporozoite maturation (Modrzynska et al., 2017). These rather conflicting findings may be explained by functional divergence of AP2-SP3/AP2-Tel in different malaria parasites. Consistent with this hypothesis, we found that *ap2-sp3/ap2-tel* is marked by HP1 in *P*. *vivax* and the three species infecting rodents, but not in *P*. *falciparum* and *P*. *knowlesi*. Another interesting HP1 target gene encodes a putative CCCH-type ZnF protein in *P*. *vivax* (PVP01_0604500) and *P*. *knowlesi* (PKNH_0603500). Proteins carrying these domains typically bind RNA and control gene expression by regulating mRNA turnover (Fu and Blackshear, 2017). Hence, this factor may act in a similar way to regulate important processes specifically in the *P*. *vivax*/*P*. *knowlesi* clade, and it will be interesting to find out in which life cycle stage(s) this may take effect.

A previous study reported a substantial degree of variegated gene expression in *P*. *falciparum* and showed that most genes affected are located in heterochromatin (Rovira-Graells et al., 2012). Their results also suggested that the transcriptional states of individual genes are stably inherited during *in vitro* culture. Our results are consistent with these findings. First, up to one-third of all heterochromatic genes showed variable PfHP1 occupancy between strains, which likely contributes to differential gene expression. Second, we observed a complete lack of variation in PfHP1 occupancy between the different IDC stages and found only 12 differentially marked genes between two clones of *P*. *knowlesi* that have been cultured independently in RBCs from two different hosts for over 200 generations (Moon et al., 2013). Together, these findings suggest that heterochromatin is faithfully maintained and that heritable changes occur rather infrequently during asexual proliferation *in vitro*.

In many multicellular eukaryotes, epigenetic mechanisms are employed in a developmental context to progressively silence groups of genes no longer required in differentiated cells (Becker et al., 2016). We found that in a somewhat analogous fashion, many genes display altered HP1 occupancy between asexually reproducing and sexually differentiating parasites. Importantly, since these cell populations were generated from the same strain in one continuous *in vitro* culture experiment, the observed changes directly reflect the dynamics of heterochromatin restructuring associated with the cell fate switch. Besides *ap2-g*, a few other early gametocyte-specific genes already showed reduced HP1 occupancy in stage II/III gametocytes, suggesting that their derepression occurred alongside that of *ap2-g* during sexual commitment or in the subsequent gametocyte ring stages. On the contrary, a larger group of genes devoid of HP1 in schizonts became heterochromatinized during gametocyte differentiation. Strikingly, many of these genes encode RBC remodeling factors. The most compelling example is related to the knob structures, parasite-induced aggregates underneath the erythrocyte membrane that are crucial for the PfEMP1-dependent adherence of iRBCs to endothelial cells and their consequent sequestration in the microvasculature (Boddey and Cowman, 2013). Although stage I to IV gametocytes also sequester, primarily in the bone marrow (Joice et al., 2014), their cytoadhesive properties are markedly different and reflected in the absence of knobs in gametocyte-infected erythrocytes (Sinden, 1982, Tiburcio et al., 2013). Our findings suggest that the mechanism responsible for preventing expression of these structures in gametocytes is based on HP1-dependent silencing of *kahrp* and other genes linked to knob formation. Given that many additional genes implicated in host cell remodeling also become associated with HP1 in gametocytes, we speculate that *P*. *falciparum* gametocytes use heterochromatin spreading as a general mechanism to inactivate host cell remodeling machinery that is crucial for the survival of asexual parasites but incompatible with the distinct biology of differentiating gametocytes.

Qualitative comparison between our data and genome-wide H3K9me3 ChIP-seq profiles obtained from *P*. *falciparum* oocysts and salivary gland sporozoites (Gomez-Diaz et al., 2017) suggests that further expansion of heterochromatic domains in these life cycle stages might lead to silencing of yet another set of genes during development in the mosquito (Figure S5). Collectively, our findings reveal that distinct changes in heterochromatin organization accompany developmental stage transitions during parasite transmission, reflecting the different biology, environmental niches, and requirements for rapid adaptive responses associated with each life cycle stage. Such silencing mechanisms must be reversed at some point to enable re-expression of affected genes during the life cycle stages wherein their expression is required. Two recent studies provided evidence that epigenetic reprogramming during mosquito passage may reset virulence gene expression in *P*. *chabaudi* (Brugat et al., 2017, Spence et al., 2013). Based on our results, HP1 likely plays a central role in such a process, and it will be interesting to build on the tools and knowledge generated here to investigate this intriguing possibility in more detail.

In conclusion, we demonstrate that the HP1-dependent silencing of genes implicated in antigenic variation, invasion, or sexual conversion is evolutionarily conserved in malaria parasites. We further identify a number of genes that are marked by HP1 specifically in one or a few species only. These may play crucial roles in the adaptive control of species- or clade-specific processes. Our results also reveal that gametocyte differentiation is accompanied by changes in heterochromatin distribution that potentially affect the expression of more than 100 genes. This raises the exciting possibility that despite their large evolutionary distance, malaria parasites employ a strategy similar to that of metazoans to regulate expression of cell-type-specific genes via heterochromatinization.

## STAR★Methods

### Key Resources Table

**Table:** 

REAGENT or RESOURCE	SOURCE	IDENTIFIER
**Antibodies**		

Rabbit polyclonal anti-*Plasmodium falciparum* HP1	Brancucci et al., 2014	N/A
Rabbit polyclonal anti-*Plasmodium berghei* HP1	This paper	N/A
Rabbit polyclonal anti-*Plasmodium vivax* HP1	This paper	N/A
Alexa Fluor 488-conjugated Goat anti-Rabbit IgG	ImmunoJackson	Cat#111-545-003; RRID: AB_2338046
Alexa Fluor 488-conjugated Goat anti-Rabbit IgG	Thermo Fisher Scientific	Cat#A-11008; RRID: AB_143165

**Bacterial and Virus Strains**		

*E*. *coli* Rosetta 2 (DE3)	EMD Millipore	Cat#71397-3

**Biological Samples**		

*Plasmodium vivax* clinical isolates	Shoklo Malaria Research Unit, Thailand	N/A
*Plasmodium vivax* thin blood smear	Swiss TPH / PNGIMR	N/A

**Chemicals, Peptides, and Recombinant Proteins**		

*Plasmodium berghei* HP1	This paper	N/A
*Plasmodium vivax* HP1	This paper	N/A
Dynabeads Protein A	Thermo Fisher Scientific	Cat#10008D
Dynabeads Protein G	Thermo Fisher Scientific	Cat#10009D
Nycodenz	Axis-Shield	Cat#1002424
Formaldehyde solution 36.5-38%	Sigma-Aldrich	Cat#F8775
ProLong Antifade mountant with DAPI	Thermo Fisher Scientific	Cat#P36931
VECTASHIELD mounting medium containing DAPI	Vector Laboratories	Cat#H-1200
cOmplete™, Mini Protease Inhibitor Cocktail	Sigma-Aldrich	Cat#11836153001

**Critical Commercial Assays**		

NEXTflex® ChIP-Seq Barcodes - 24	Bio Scientific	Cat#NOVA-514122
KAPA HiFi HotStart ready mix	KAPA Biosystems	Cat#KM2602
Agencourt AMPure XP	Beckman Coulter	Cat#A63880
NextSeq 500/550 High Output v2 kit (75 cycles)	Illumina	Cat#FC-404-2005

**Deposited Data**		

ChIP-Seq data	This paper	GEO: GSE102695
Pf2004 draft genome assembly	Pf3K consortium and the Wellcome Trust Sanger Institute	ftp://ftp.sanger.ac.uk/pub/project/pathogens/Plasmodium/falciparum/PF3K/SecondSetReferenceGenomes/DraftAnnotation/Pf2004/.

**Experimental Models: Cell Lines**		

Parasite strain: *Plasmodium falciparum* 3D7	Walliker et al., 1987	Alan Cowman, WEHI, Melbourne, Australia
Parasite strain: *Plasmodium falciparum* Pf2004/164tdT	Brancucci et al., 2015	N/A
Parasite strain: *Plasmodium falciparum* NF54	Delemarre and van der Kaay, 1979	Robert Sauerwein, Radboudumc, Nijmegen, NL
Parasite strain: *Plasmodium falciparum* NF135	Teirlinck et al., 2013	Robert Sauerwein, Radboudumc, Nijmegen, NL
Parasite strain: *Plasmodium knowlesi* A1-H.1	Moon et al., 2013	Mike Blackman, Francis Crick Institute, London, UK
Parasite strain: *Plasmodium knowlesi* A1-C.1	Moon et al., 2013	Mike Blackman, Francis Crick Institute, London, UK
Parasite strain: *Plasmodium berghei* ANKA	BEI Resources (MR4)	N/A
Parasite strain: *Plasmodium chabaudi chabaudi* AS	Cross and Langhorne, 1998	Jean Langhorne, Francis Crick Institute, London
Parasite strain: *Plasmodium yoelii yoelii* YM	BEI Resources (MR4)	N/A

**Experimental Models: Organisms/Strains**		

Mouse: BALB/c (female)	In Vivos Pte Ltd. Singapore	NA

**Oligonucleotides**		

Pb_F: aaaagatttcatatgacaggatcagatg	This paper	N/A
Pb_R: ttccctcgagcaccgttctatatctaagtc	This paper	N/A
SUMO_F: tttcatatgcatcatcatcatcatcacgggtcggactcag aagtcaatc	This paper	N/A
SUMO_R: cctaggatccggcgccaccaatctgttctctgtg	This paper	N/A
Pv_F: actggatccgatgaagagtttgaaatagg	This paper	N/A
Pv_R: tgtgctcgagtacttaggccgttcggtatcg	This paper	N/A

**Recombinant DNA**		

pET20b(+)	EMD Millipore	Cat#69739-3
pET_PbHP1-6xHis	This paper	N/A
pETA-HS	This paper	N/A
pETA-HS-PvHP1	This paper	N/A
pETA-Strep	This paper	N/A
pETA_Strep-PvHP1-6xHis	This paper	N/A

**Software and Algorithms**		

Clustal Omega	Sievers et al., 2011	https://www.ebi.ac.uk/Tools/msa/clustalo/
MEGA7	Kumar et al., 2016	http://www.megasoftware.net/download_form
Olympus DP manager software (v2.2.1.195)	Olympus	N/A
ImageJ	Schneider et al., 2012	https://imagej.net/Downloads
Nikon Elements Advanced Research	Nikon	N/A
Leica IM1000 software	Leica	N/A
Fiji	Schindelin et al., 2012	https://imagej.net/Fiji
jvenn	Bardou et al., 2014	http://jvenn.toulouse.inra.fr/app/index.html
BWA (v0.7.12-r1039)	Li and Durbin, 2009	https://insidedna.me/tool_page_assets/pdf_manual/bwa.pdf
SAMtools (v1.2)	Li et al., 2009	http://www.htslib.org/download/
BEDTools (v2.20.1)	Quinlan and Hall, 2010	http://bedtools.readthedocs.io/en/latest/content/installation.html
SignalMap Software v2.0	Roche	http://sequencing.roche.com/en/products-solutions/by-category/target-enrichment/software/signal-map-software.html
UCSC Genome Browser	UCSC Genome Browser	https://genome-store.ucsc.edu/
Rstudio (v3.3.2)	RStudio	https://www.rstudio.com/products/rstudio/download/

**Other**		

Plasmodipure filters	EuroProxima	Cat#8011Filter25u
HisTrap HP	GE Healthcare	Cat#17-5248-01
HiTrap Protein A HP	GE Healthcare	Cat#17-0403-01
HiTrap NHS activated HP column	GE Healthcare	Cat#17-0716-01
StrepTrap HP	GE Healthcare	Cat#28-9136-30
Amicon Ultra Centrifugal Filter 10KDa	EMD Millipore	Cat#UFC801024
E-Gel Size Select agarose gel	Thermo Fisher Scientific	Cat#G661012
Non-woven fabric filters	ZXBio.net	N/A
Glycerolyte Solution 57	Fenwal	Cat#FWL4A7831
BD Vacutainer™ Plastic Blood Collection Tubes with Sodium Heparin: Conventional Stopper	Fisher Scientific	Cat#BD367874
BD Vacutainer™ Plastic Blood Collection Tubes with K2EDTA: Tube Stopper	Fisher Scientific	Cat#BD367844

### Contact for Reagent and Resource Sharing

Further information and requests for reagents should be directed to and will be fulfilled by the Lead Contact, Till S. Voss (till.voss@swisstph.ch).

### Experimental Model and Subject Details

#### Mouse Model

Mice used in this study (BALB/c mice; age 6-8 weeks; weight 25-30 g) were maintained in accordance with the NACLAR (National Advisory Committee for Laboratory Animal Research) guidelines under the Animal & Birds (Care and Use of Animals for Scientific Purposes) Rules of Singapore with approval from the Institutional Animal Care and Use Committee (IACUC) of Nanyang Technological University (NTU) of Singapore (Approval number: ARFSBS/NIE A002). All animals used in this study were obtained from InVivos Pte Ltd and subsequently housed under SPF conditions at NTU. *P*. *berghei*, *P*. *chabaudi* and *P*. *yoelii* infections for obtaining parasites for chromatin extraction were performed on female BALB/c mice (age 6-8 weeks; weight 25-30g). Mice were infected with an initial inoculum of 5x10^5^ parasites and were exsanguinated by cardiac puncture when parasitaemia levels reached between 10-20%.

#### *P*. *falciparum* Parasites

*P*. *falciparum* parasites were cultured at 37°C at 5% haematocrit based on the original protocol published by Trager and Jensen (Trager and Jensen, 1978). Growth synchronization was achieved by repeated sorbitol treatments (Lambros and Vanderberg, 1979). 3D7 parasites were cultivated with AB^+^ human RBCs in RPMI 1640/25 mM Hepes standard culture medium supplemented with 0.5% Albumax II. NF54 and NF135 parasites were cultivated with O^+^ human RBCs in RPMI 1640/25 mM Hepes standard culture medium supplemented with 10 % human serum. Pf2004/164tdTom parasites (Brancucci et al., 2015) were cultivated with AB^+^ human RBCs in RPMI 1640/25 mM Hepes standard culture medium supplemented with 10 % human serum. Pf2004/164tdTom gametocytes were generated by inducing sexual commitment as described (Brancucci et al., 2015). After re-invasion cultures were treated with 50mM N-acetylglucosamine (Fivelman et al., 2007) for three consecutive days to eliminate asexual parasites.

#### *P*. *knowlesi* Parasites

*P*. *knowlesi* A1-H.1 parasites were grown at 37°C in O+ human RBCs obtained from the United Kingdom National Blood Transfusion Service. *P*. *knowlesi* A1-C.1 parasites were grown in *M*. *fascicularis* blood provided by NIBSC (UK), which was collected by venous puncture into K2 EDTA BD Vacutainers (Fisher Scientific) as described previously (Moon et al., 2013). Samples of *M*. *fascicularis* blood used for parasite culture were provided by the National Institute for Biological Standards and Control. The rationale and procedures for venepuncture and blood sample collection were reviewed by the local Animal Welfare and Ethical Review Body (the Institutional Review Board) of the National Institute for Biological Standards and Control and performed under licence (PPL70/8506) granted by the United Kingdom Home Office as governed by United Kingdom law under the Animals (Scientific Procedures) Act 1986. Animals were handled in strict accordance with the “Code of Practice Part 1 for the housing and care of animals (21/03/05)” available at https://www.gov.uk/research-and-testing-using-animals. The work also met the National Centre for the Replacement Refinement and Reduction of Animals in Research (NC3Rs) guidelines on primate accommodation, care, and use (https://www.nc3rs.org.uk/non-human-primate-accommodation-care-and-use), which exceed the legal minimum standards required by the United Kingdom Animals (Scientific Procedures) Act 1986, associated Codes of Practice, and the US Institute for Laboratory Animal Research Guide. Parasite cultures were synchronised by centrifugation through a density cushion of Nycodenz (Axis-Shield) as previously described (Moon et al., 2013).

#### *P*. *vivax* Parasites

The clinical *P*. *vivax* isolates examined in this study were collected under the following approved ethical guidelines and protocols: OXTREC 45-09 and OXTREC 17-11 (Centre for Clinical Vaccinology and Tropical Medicine, University of Oxford, Oxford, UK), MUTM 2008-215 from the Ethics committee of the Faculty of Tropical Medicine (Mahidol University, Bangkok, Thailand) and MRAC No. 16.01 from the Medical Research Advisory Committee of Papua New Guinea. To obtain *P*. *vivax* parasites for chromatin extraction eight clinical isolates were collected from malaria patients attending clinics run by the Shoklo Malaria Research Unit, Mae Sot, Thailand. Five milliliters of whole blood were collected in lithium heparin collection tubes by venepuncture from each patient using Sodium Heparin BD Vacutainers (Fisher Scientific). After leukocyte depletion using non-woven fabric filters (ZXBio.net) these samples were cryopreserved in glycerolyte 57 solution (Fenwal) and stored in liquid nitrogen (Borlon et al., 2012). Frozen samples were thawed using a series of NaCl gradients (12%, 1.6% and 0.9%) and matured *ex vivo* for 40 hours as described (Borlon et al., 2012, Russell et al., 2011).

### Method Details

#### *P*. *falciparum* Sample Collection and Chromatin Preparation

Chromatin from 3D7 ring stages (8-16 hpi), trophozoites (32-30 hpi) or schizonts (40-48 hpi) (approximately 0.75-1.5x10^9^ parasites each) and from Pf2004/T164dTom schizonts, stage II/III gametocytes (day four after re-invasion) or stage IV/V gametocytes (day nine after re-invasion) (approximately 2x10^8^ parasites each) was prepared by crosslinking cultures with 1% formaldehyde (Sigma-Aldrich) for 15 min at 37°C. Crosslinking reactions were quenched by 0.125 M glycine. Nuclei were isolated by releasing parasites from iRBCs using 0.05% saponin followed by lysis in CLB (20 mM Hepes, 10 mM KCl, 1 mM EDTA, 1 mM EGTA, 0.65% NP-40, 1mM DTT, 1x protease inhibitor (Sigma-Adrich), pH 7.9). Nuclei were washed and snap-frozen in CLB supplemented with 50% glycerol. Chromatin from NF54 and NF135 schizont stages (approximately 2-4x10^9^ parasites each) was prepared by passing the cultures through Plasmodipure filters (EuroProxima) to remove white blood cells prior to formaldehyde crosslinking for 10 min at 37°C and quenching in 0.125 M glycine. Nuclei were isolated by releasing parasites from iRBCs using 0.05% saponin followed by gentle homogenisation (pestle B, 15 strokes) in CLB2 (10 mM Tris-HCl, 3 mM MgCl_2_, 0.2% NP40, 1x protease inhibitor (Sigma-Adrich), pH 8.0) and centrifugation through a 0.25 M sucrose cushion (in CLB2) at 2000 rpm for 10 min at 4°C. Nuclei were snap-frozen in CLB2 supplemented with 20% glycerol. Frozen nuclei were thawed and resuspended in sonication buffer (50mM Tris-HCl, 1% SDS, 10mM EDTA, 1x protease inhibitor (Sigma-Adrich), pH 8.0) and sonicated for 20-24 cycles of 30 sec ON/30 sec OFF (setting high, Bioruptor^TM^ Next Gen, Diagenode). Chromatin fragment sizes ranged from 100-600 bp as determined by de-crosslinking a 50 μl aliquot and running the purified DNA on a 1% agarose gel.

#### *P*. *knowlesi* Sample Collection and Chromatin Preparation

Cultures containing a schizont parasitaemia of around 5% were passed through Plasmodipure filters (EuroProxima) to remove white blood cells prior to crosslinking for 10 min at 37°C in 1% formaldehyde (Sigma-Aldrich) and quenching in 0.125 M glycine. Red blood cells were removed by lysis on ice for 10 min with 0.15% saponin/PBS before washing the pellet in PBS and snap-freezing the parasite pellets in liquid nitrogen. Nuclei were isolated by lysis in CLB, washed and aliquots corresponding to approximately 1x10^9^ nuclei were snap-frozen in CLB supplemented with 50% glycerol. Preparation of sheared chromatin was performed as described above for *P*. *falciparum*.

#### *P*. *berghei*, *P*. *chabaudi* and *P*. *yoelii* Sample Collection and Chromatin Preparation

For each of the three parasite species, whole blood of infected mice containing approximately 5x10^9^ schizonts (three mice for *P*. *yoelii yoelii* YM, four mice for *P*. *chabaudi chabaudi* AS, five mice for *P*. *berghei* ANKA) was diluted in standard *P*. *falciparum* culture medium and passed through a Plasmodipure filter (EuroProxima) to remove white blood cells. The purified RBCs were collected by centrifugation at 2’000 rpm for 5 min, resuspended in 30 ml culture medium and crosslinked at 37°C for 10 min in presence of 1% formaldehyde (Sigma-Aldrich). Crosslinking reactions were quenched by 0.125 M glycine. The crosslinked RBCs suspension was split into three equal aliquots, centrifuged at 2’000 rpm for 5 min, supernatants were removed and the RBC pellets snap-frozen in liquid nitrogen. Nuclei were isolated by releasing parasites from iRBCs using 0.05% saponin followed by lysis in CLB (20 mM Hepes, 10 mM KCl, 1 mM EDTA, 1 mM EGTA, 0.65% NP-40, 1mM DTT, 1x protease inhibitor (Sigma-Adrich), pH 7.9). Again, nuclei were washed and aliquots corresponding to approximately 1x10^9^ nuclei were snap-frozen in CLB supplemented with 50% glycerol. Preparation of sheared chromatin was performed as described above for *P*. *falciparum*.

#### *P*. *vivax* Sample Collection and Chromatin Preparation

The *P*. *vivax ex vivo* schizont cultures were crosslinked at 37°C for 10 min in presence of 1% formaldehyde (Sigma-Aldrich) and subsequently the reactions were quenched by 0.125 M glycine. The crosslinked RBCs were centrifuged at 200 g for 5 min, supernatants were removed and the RBC pellets snap-frozen in liquid nitrogen. The eight samples were thawed and pooled and nuclei isolated by releasing parasites from iRBCs using 0.05% saponin followed by lysis in CLB. Nuclei were washed and snap-frozen in CLB supplemented with 50% glycerol. Preparation of sheared chromatin was performed as described above for *P*. *falciparum*.

#### Phylogenetic Analysis of *Plasmodium* HP1 Orthologs

Protein sequences of *Plasmodium* HP1 orthologs were downloaded from PlasmoDB v33 and used to perform a multiple sequence alignment using Clustal Omega (Sievers et al., 2011) with default parameters. Phylogenetic tree construction was done with MEGA7 (Kumar et al., 2016) using the Neighbor-joining method and 1’000 bootstrap replicates.

#### Generation and Affinity Purification of α-PbHP1 and α-PvHP1 Antibodies

All recombinant proteins were expressed in Rosetta2(DE3) cells (EMD Millipore) using auto-induction (Studier, 2005). The sequence encoding PbHP1 was amplified from gDNA using primers Pb_F (aaaagatttcatatgacaggatcagatg) and Pb_R (ttccctcgagcaccgttctatatctaagtc) and cloned into pET20b(+) (EMD Millipore) using *Nde*I and *Xho*I restriction sites in order to express PbHP1 fused to a C-terminal 6xHis tag (pET_PbHP1-6xHis). Recombinant PbHP1-6xHis was purified using a HisTrap HP column (GE Healthcare) using buffer NiB (50 mM H_3_PO_4_, 0.5 M NaCl, 20 mM imidazole, pH 7.4) supplemented with 8 M urea for lysis, binding and washing, and buffer NiE (50mM H_3_PO_4_, 0.5 M NaCl, 225 mM imidazole, pH 7.4) containing 8 M urea for elution. The elution was diluted 1:4 with H_2_O and the eluted proteins were precipitated with trichloroacetic acid (TCA). PvHP1 was expressed as an N-terminally tagged 6xHis-SUMO fusion protein (HS-PvHP1). The parental expression vector pETA-HS was generated by introducing a sequence encoding a fusion tag consisting of a 6xHis stretch followed by *Saccharomyces cerevisiae* SUMO, amplified from gDNA using primers SUMO_F (tttcatatgcatcatcatcatcatcacgggtcggactcagaagtcaatc) and SUMO_R (cctaggatccggcgccaccaatctgttctctgtg) between the *Nde*I and *BamH*I sites of pET20(b)+ (EMD Millipore), yielding a vector similar to the one described by Malakhov and colleagues (Malakhov et al., 2004). The *pvhp1* insert was cloned into pETA-HS by ligating a *BamH*I/*Xho*I-digested PCR product amplified from gDNA using primers Pv_F (actggatccgatgaagagtttgaaatagg) and Pv_R (tgtgctcgagtacttaggccgttcggtatcg) (pETA-HS-PvHP1). HS-PvHP1 was purified using a HisTrap HP column (GE Healthcare) and buffers NiB for lysis, binding and washing, and buffer NiE for elution. The elution was subject to buffer exchange with NiB supplemented with 1 mM Tris-(2-carboxyethyl)-phosphin (TCEP) and the HS-PvHP1 fusion protein was digested using recombinant SUMO protease (L403-K621 of *S*. *cerevisia* ULP1; GB1-ULP1-6xHis). The cleaved tag and SUMO protease were subtracted using a HisTrap HP column (GE Healthcare) and the purified untagged PvHP1 protein was precipitated with TCA.

Purified recombinant PbHP1-6xHis and untagged PvHP1 were used to immunize rabbits (Pacific Immunology). Total rabbit IgG from anti-PbHP1 and anti-PvHP1 immune sera were purified using HiTrap Protein Protein A HP columns (GE Healthcare) using a mild arginine elution method similar to the one described by Arakawa and colleagues (Arakawa et al., 2004), with the exception that a linear combined pH and arginine gradient elution was used instead of stepwise elution. Rabbit sera were diluted 1:3 using buffer IgGA (750 mM L-arginine, 150 mM H_3_PO_4_, 150 mM citric acid, pH 7.3), bound to 5 ml HiTrap Protein A HP columns (GE Healthcare) and washed with five column volumes (CVs) of buffer IgGA. Antibodies were eluted using a linear gradient (eight CVs) of buffers IgGA to IgGB (2 M L-arginine, 150 mM H_3_PO_4_, 150 mM citric acid, adjusted to pH 3.7 using HCl). The antibodies eluted in a symmetrical peak (maximum at 1.4 M arginine and pH 5). Purified antibodies were subject to buffer exchange with PBS.

Affinity purification of α-PbHP1 antibodies was done as previously described for α-PfHP1 antibodies (Brancucci et al., 2014) with the exception that the PbHP1-6xHis antigen was bound to the nickel column in buffer NiB containing 2 M urea. For the PvHP1 antigen, we first generated the parental pETA_Strep vector facilitating expression of N-terminally Strep(II)-tagged and C-terminally 6xHis-tagged fusion proteins by replacing the *Nde*I/*BamH*I fragment in pET20b(+) with an annealed double-stranded oligonucleotide (Strep_F (tatggctagctggagccacccgcagttcgaaaaag) and Strep_R (gatcctttttcgaactgcgggtggctccagctagcca)) encoding Met-Ala-Ser-Strep(II). Next, the same PCR product that was used to generate HS_PvHP1 (see above) was cloned into the pETA_Strep vector using *BamH*I and *Xho*I to obtain pETA_Strep-PvHP1-6xHis. The Strep(II)-PvHP1-6xHis fusion protein was purified using a HisTrap HP column (GE Healthcare) and the same buffers as used for purification of HS-PvHP1 (see above) followed by a StrepTrap HP column (GE Healthcare) using buffer NiB containing 1 mM EDTA for washing or 2.5 mM desthiobiotin for elution. The purified protein was subject to buffer exchange with coupling buffer (0.2 M NaHCO_3_, 0.5 M NaCl, pH 8.3). Next, the protein was coupled to a HisTrap NHS-activated HP column (GE Healthcare) following the supplier’s instructions. α-PvHP1 IgG was diluted 1:5 in buffer IgGA and bound to the Strep(II)-PvHP1-6xHis column. The column was washed with 20 CVs of buffer IgGA and eluted with buffer IgGB. Both, α-PvHP1 and α-PbHP1 antibodies were finally subject to buffer exchange with PGS (20 mM H_3_PO_4_, 30 mM KOH, 25% glycerol, 250 mM Na_2_SO_4_, pH 6.8) and concentrated to 0.4-0.7 mg/ml using an Amicon Ultra spin filter with a 10K cutoff (EMD Millipore).

#### Fluorescence Microscopy

IFAs for *P*. *falciparum* were performed as described previously (Brancucci et al., 2014). IFAs for *P*. *berghei*, *P*. *chabaudi* and *P*. *yoelii* were performed with acetone:methanol (9:1)-fixed cells using rabbit α-PbHP1 (1:250) and Alexa Fluor 488-conjugated α-rabbit IgG (1:500) (ImmunoJackson). Slides were viewed under Olympus IX71 fluorescence microscope using a 100x oil immersion objective and equipped with an Olympus DP30BW camera. Images were acquired via the Olympus DP manager software (v2.2.1.195) and processed using ImageJ (v1.440) (Schneider et al., 2012). IFAs for *P*. *knowlesi* were performed using blood smears fixed with 4% paraformaldehyde for 30 min followed by three washes in PBS and permeabilisation in 0.1% Triton-X100 for 10 min. Slides were blocked overnight at 4°C in 3% BSA/PBS and then labelled with rabbit α-PvHP1 (1:600) and Alexa Fluor 488-conjugated α-rabbit IgG (1:5’000) (Thermo Fisher Scientific). The smear was mounted in ProLong Antifade mountant with DAPI (Thermo Fisher Scientific). Slides were viewed with a Nikon Ti E inverted microscope using a 100x oil immersion objective and imaged with an ORCA Flash 4.0 CMOS camera (Hamamatsu). Images were acquired and processed using the Nikon Elements Advanced Research software package. IFAs for *P*. *vivax* were performed using methanol-fixed thin blood smears. Slides were blocked using 3% BSA/PBS and then labeled with α-PvHP1 (1:500) and Alexa Fluor 488- conjugated α-rabbit IgG (1:500) (Thermo Fisher Scientific) antibodies in 3% BSA/PBS. Slides were mounted using VECTASHIELD mounting medium containing DAPI (Vector Laboratories). Images were taken at 100-fold magnification on a Leica DM 5000B microscope with a Leica DFC 345 FX camera, acquired via the Leica IM1000 software, and processed using Fiji (Schindelin et al., 2012). For each experiment, images were acquired and processed with identical settings.

#### Western Blot

*P*. *berghei*, *P*. *chabaudi* and *P*. *yoelii* schizonts were enriched using a 50-60% Histodenze (Sigma-Aldrich) gradient. Parasites were released from iRBCs by saponin lysis, resuspended in Urea extraction buffer (EMD Millipore) and separated by SDS-PAGE. Proteins were detected using rabbit α-PbHP1 (1:2’000) antibodies. *P*. *knowlesi* A1-H.1 schizonts were enriched on a density cushion of 55% Nycodenz (Axis-Shield) stock solution (27.6% (wt/vol) Nycodenz in 10 mM Hepes, pH 7.0) in RPMI-1640 medium. RBCs were lysed using 0.15% saponin/PBS and the resultant parasite pellet was diluted 1:100 in Urea extraction buffer (40 mM Tris-HCl, 1 mM EDTA, 8 M Urea, 5% SDS, 1x protease inhibitor cocktail (Sigma-Adrich), 1% β-mercaptoethanol), mixed with SDS sample buffer and separated by SDS-PAGE alongside a similarly treated uninfected RBC control. PkHP1 was detected with rabbit α-PvHP1 antibodies (1:5’000).

#### Chromatin Immunoprecipitation

For each ChIP reaction, sonicated chromatin containing 500 ng of DNA was incubated in incubation buffer (0.75% SDS, 5% Triton-X100, 750 mM NaCl, 5 mM EDTA, 2.5 mM EGTA, 100 mM Hepes, pH 7.4) with either 1 μg rabbit α-PfHP1 (for *P*. *falciparum*), 1 μg rabbit α-PvHP1 (for *P*. *vivax* and *P*. *knowlesi*) or 1 μg rabbit α-PbHP1 (for *P*. *berghei*, *P*. *chaubaudi* and *P*. *yoelii*) as well as 10 μl protA and 10 μl protG Dynabeads suspension (Thermo Fisher Scientific).

For each sample four ChIP reactions were prepared and incubated overnight at 4°C while rotating. Beads were washed twice with wash buffer 1 (0.1% SDS, 0.1% DOC, 1% Triton-X100, 150 mM NaCl, 1 mM EDTA, 0.5 mM EGTA, 20 mM Hepes, pH 7.4), once with wash buffer 2 (0.1% SDS, 0.1% DOC, 1% Triton-X100, 500 mM NaCl, 1 mM EDTA, 0.5 mM EGTA, 20 mM Hepes, pH 7.4), once with wash buffer 3 (250 mM LiCl, 0.5% DOC, 0.5% NP-40, 1 mM EDTA, 0.5 mM EGTA, 20 mM Hepes, pH 7.4) and twice with wash buffer 4 (1 mM EDTA, 0.5 mM EGTA, 20 mM Hepes, pH 7.4). Each wash was performed for 5 min at 4°C while rotating. Subsequently, immunoprecipitated chromatin was eluted in elution buffer (1% SDS, 0.1M NaHCO_3_) at room temperature. The eluted chromatin samples and the corresponding input samples (sonicated input chromatin containing 500 ng DNA) were de-crosslinked in 1% SDS/0.1 M NaHCO_3_/1 M NaCl at 45°C overnight while shaking. For each parasite strain or species the separate ChIP samples were combined and the DNA was purified using QIAquick MinElute PCR columns (Qiagen).

#### High-Throughput Sequencing

For each sequencing library 2-10 ng of ChIP or input DNA were end-repaired, extended with 3′ A-overhangs and ligated to barcoded NextFlex adapters (Bio Scientific) as described previously (Hoeijmakers et al., 2011). Libraries were amplified (98°C for 2 min; four cycles 98°C for 20 sec, 62°C for 3 min; 62°C for 5 min) using KAPA HiFi HotStart ready mix (KAPA Biosystems) and NextFlex primer mix (Bio Scientific) as described (Kensche et al., 2016). 225-325 bp fragments (including the 125 bp NextFlex adapter) were size-selected using a 2% E-Gel Size Select agarose gel (Thermo Fisher Scientific) and amplified by PCR for eight or ten cycles (Table S7) under the same condition as described above. Library purification and removal of adapter dimers was performed with Agencourt AMPure XP beads in a 1:1 library:beads ratio (Beckman Coulter). ChIP-seq libraries were sequenced for 75 bp single-end reads using the NextSeq 500/550 High Output v2 kit (Illumina) on the Illumina NextSeq 500 system.

### Quantification and Statistical Analysis

#### High-Throughput Sequencing Data Analysis

Using BWA samse (v0.7.12-r1039) (Li and Durbin, 2009) sequencing reads were mapped against the respective reference genomes available on PlasmoDB v26, namely *P*. *berghei* ANKA, *P*. *chabaudi chabaudi*, *P*. *yoelii yoelii* YM, *P*. *falciparum* 3D7, *P*. *knowlesi* H and *P*. *vivax* P01 (PlasmoDB v29). Reads from the *P*. *falciparum* Pf2004 ChIP-seq libraries were additionally mapped against a Pf2004 genome assembly, which was obtained after long-read PacBio sequencing in the framework of the Pf3K reference project.

Mapped reads were filtered to mapping quality ≥15 (SAMtools v1.2) (Li et al., 2009) and only uniquely mapped reads (3.4-22 million reads for α-HP1 ChIP samples and 6.5-55 million reads for input samples) were used for further analysis (Table S7). ChIP-seq data were visualized in the UCSC Genome browser (https://genome-store.ucsc.edu/). All libraries were normalized to the number of mapped reads per million (RPM) and bedgraph files were generated using BEDtools (v2.20.1) (Quinlan and Hall, 2010). For log2 ratio tracks α-HP1 ChIP values were divided by input values and log2-transformed using BEDtools (v2.20.1) (Quinlan and Hall, 2010). Within the UCSC genome browser tracks were smoothened and the windowing function was set as ‘mean’.

To calculate the HP1 coverage for individual genes, tags were counted in a 1000 bp window (ATG ± 500 bp) for each coding sequence and offset by +1 to avoid division by zero while calculating fold changes in coverage. α-HP1 ChIP-seq and input tag counts were normalized to the number of reads per kb per million mapped reads (RPKM). ChIP-seq enrichment values were calculated as α-HP1 ChIP [RPKM]/input [RPKM]. Genes encoded by the mitochondrial or apicoplast genomes and nuclear genes with low mappability (input RPKM < 5) were excluded from downstream analysis.

To visualize the genome-wide HP1 coverage in schizont stages of the six *Plasmodium* species (*P*. *berghei*, *P*. *yoelii*, *P*. *chaubaudi*, *P*. *knowlesi*, *P*. *vivax*, *P*. *falciparum*) the respective reference genomes were divided into 1000 bp windows using BEDtools (v2.20.1) (Quinlan and Hall, 2010). For each window ChIP-seq enrichment values were calculated as described above, log2-transformed and visualized using the software SignalMap v2.0 (www.sequencing.roche.com). Windows with less than five tag counts in the ChIP-seq and/or the input sample were set to '0' and defined as regions with low mappablity. To identify and compare the sets of heterochromatic genes across the six *Plasmodium* species ChIP-seq enrichment values were calculated and assigned to either a ‘heterochromatic’ or ‘euchromatic’ compartment. To do so, we fitted a bivariate Gaussian mixture model to the data and calculated the probabilities (p) for genes to belong to either one of the two compartments using the modelling tool ‘normalmixEM‘ from the R package ‘mixtools’. For further analysis genes with p > 0.99999 for the ‘heterochromatic’ compartment were considered high confidence heterochromatic genes. Genes with 0.99999 > p > 0.95 were considered potential heterochromatic genes and genes with p < 0.95 were placed in the ‘euchromatic’ compartment. To look for orthologs and syntenic orthologs, high confidence heterochromatic genes (p > 0.99999) for each species were imputed into PlasmoDB v33. For instance, the orthologs (syntenic/non-syntenic) for *P*. *falciparum* heterochromatic genes (403 genes) were transformed into orthologs of *P*. *berghei* ANKA, *P*. *yoelii yoelii* YM, *P*. *chaubaudi chabaudi*, *P*. *knowlesi* strain H and *P*. *vivax* P01 using the function ‘transform by orthology’ in PlasmoDB. Similarly, the orthologs for heterochromatic genes in *P*. *vivax* (834 genes), *P*. *knowlesi* (355 genes), *P*. *berghei* (192 genes), *P*. *chabaudi* (369 genes) and *P*. *yoelii* (907 genes) were individually transformed into orthologs of the five other *Plasmodium* species. Ortholog sets (syntenic/non-syntenic) among the species were identified using jvenn (Bardou et al., 2014) and assigned according to the species identifier pf, pv, pk, pb, pc or py (Tables S1 and S2).

To investigate the association between heterochromatic region and distribution of *kir* and *SICAvar* genes as well as interstitial telomere repeat sequences (ITSs) PkHP1 coverage in *P*. *knowlesi* schizont stages was depicted as described above. Coding sequences of *kir*/*kir*-like and *SICAvar* genes were depicted according to their genomic coordinates within the *P*. *knowlesi* H genome (PlasmoDB v26). ITSs were identified by searching the *P*. *knowlesi* genome for occurrences of GGGTTTA or GGGTTCA repeats on both strands using regular expression. The number of these sequences were counted at every 100 bp window and windows with three or more hits were considered (imperfect repeats with mismatches were not considered). To compare PkHP1 occupancy between two clones of *P*. *knowlesi* (A1-H.1 and A1-C.1) the ratio between PkHP1 occupancy values (ChIP/Input) were calculated for each gene. Based on visual inspection of the UCSC Genome browser tracks we considered 2.5-fold difference as a marked and likely influential change in in PkHP1 occupancy. Genes with low mappability (input RPKM < 5) in at least one of the clones were excluded from downstream analyses.

To allow direct comparison of HP1 gene coverage across different *P*. *falciparum* strains (3D7, Pf2004, NF135 and NF54) PfHP1 ChIP-seq reads from all four strains were mapped against the *P*. *falciparum* 3D7 reference genome (PlasmoDB v26). ChIP-seq reads from strain Pf2004 schizonts were additionally mapped against the Pf2004 reference genome (Table S7). Note that although matching reference genomes do exist for NF54 and NF135 (Plas_falc_NF54_v1 and Plas_falc_NF135_5_C10_v1; http://protists.ensembl.org) the assembly and annotation of these reference genomes are fragmented and not sufficiently informative for analysis of heterochromatin organisation. Genes with low mappability (input RPKM < 5) in at least one of the strains were excluded from downstream analyses. For the remaining genes ChIP-seq enrichment values were z-score transformed, k-means clustered and depicted as heatmap using the R package ‘pheatmap’. To visualize the average HP1 occupancy for all strains investigated in this study (Figure 4B), the 3D7 reference genome was divided into 1000 bp windows using BEDtools (v2.20.1) (Quinlan and Hall, 2010). For each window ChIP-seq enrichment values were calculated as described above, z-score transformed, averaged across the strains and visualized using the software SignalMap v2.0 (www.sequencing.roche.com). Coding sequences of the genes in k-means clusters 5 to 11 were depicted according to their location within the *P*. *falciparum* 3D7 genome (PlasmoDB v26).

For the comparison of intra-erythrocytic stages, ChIP-seq enrichment values were calculated using ChIP RPKM values of ring stages (8-16 hpi), trophozoites (24-32 hpi) or schizonts (40-48 hpi) and input RPKM values obtained from the schizont sample. The relation between PfHP1 gene coverage and transcript abundance was visualized by sorting PfHP1 ChIP-seq enrichment values for each gene in schizont stages from high to low, plotted against the corresponding transcript abundance value (RPKM). For the latter, directional RNA-seq data from eight intra-erythrocytic stages (Kensche et al., 2016) were aligned against the annotated *P*. *falciparum* 3D7 transcriptome (PlasmoDB v26) and filtered for uniquely mapped reads and mapping quality ≥ 15. Reads were separated according to the strand they mapped to (sense strand FLAG16; antisense strand FLAG0). Reads aligning to the sense strand were used for further analysis. For each transcript (excluding mitochondrial RNA and apicoplast RNA) tags were counted, offset by +1 and normalized to the number of reads per kb per million mapped reads. For each gene the maximum transcript abundance value (RPKM) observed during intra-erythrocytic development was plotted in the scatter plot and *var* genes as well as genes displaying clonally variant expression (Rovira-Graells et al., 2012) were specifically highlighted.

To assess differences in PfHP1 occupancy between Pf2004 schizonts, stage II/III gametocytes and stage IV/V gametocytes ChIP-seq reads were mapped against the *P*. *falciparum* 3D7 genome (PlasmoDB v26) and the *P*. *falciparum* Pf2004 reference genome (Table S7). Genes with low mappability (input RPKM < 5) in at least one of the stages were excluded from downstream analysis. For the remaining genes ChIP-seq enrichment values were z-score transformed, k-means clustered and depicted as a heatmap using the R package ‘pheatmap’. To visualize the genome-wide PfHP1 occupancy for schizont stages and stage IV/V gametocytes (Figure 6C) the *P*. *falciparum* 3D7 reference genome was divided into 1000 bp windows using BEDtools (v2.20.1) (Quinlan and Hall, 2010). For each window ChIP-seq enrichment values were calculated as described above, z-score transformed and visualized using the software SignalMap v2.0 (www.sequencing.roche.com). Coding sequences of the genes in k-means clusters 5 and 6 to 8 were depicted according to their location within the *P*. *falciparum* 3D7 genome (PlasmoDB v26). Additionally, we visually compared our Pf2004 PfHP1 ChIP-seq data with the midgut oocyst and salivary gland sporozoite H3K9me3 ChIP-seq datasets generated by Gómez-Díaz and colleagues (Gomez-Diaz et al., 2017) using the UCSC genome browser (https://genome-store.ucsc.edu/). Midgut oocyst and salivary gland sporozoite H3K9me3 ChIP-seq and input data (GEO accession numbers GSM1981878, GSM1981880, GSM1981883, GSM1981885) were aligned to the *P*. *falciparum* 3D7 reference genome (PlasmoDB v26) (Table S7) and processed as described above to generate bedgraph log2 H3K9me3-ChIP/Input ratio files.

### Data and Software Availability

The accession number for the ChIP-seq data reported in this paper is GEO: GSE102695. The sequence and annotation of the Pf2004 genome is available at ftp://ftp.sanger.ac.uk/pub/project/pathogens/Plasmodium/falciparum/PF3K/SecondSetReferenceGenomes/DraftAnnotation/Pf2004/.

## Supplemental Information

Document S1. Figures S1–S5

Table S1. Genome-wide HP1 Occupancy in Six *Plasmodium* Species, Related to Figures 1 and 2 and Tables S2–S5 and S7 ChIP/input enrichment values were calculated over a 1,000-bp window (ATG ±500 bp) for each coding sequence of the respective reference genomes in *P*. *falciparum*, *P*. *vivax*, *P*. *knowlesi*, *P*. *chabaudi*, *P*. *berghei*, and *P*. *yoelii* schizont stages. Column A: chromosome number. Columns B and C: nucleotide positions at the start and end of each 1,000 bp window. Columns D and E: gene IDs and annotations. Columns F and G: RPKM values for the α-ChIP and input samples. Column H: HP1 enrichment values (ChIP/input ratio). Columns I and J: probability values (p) for genes to belong to either the euchromatic or the heterochromatic group of genes. Column K: heterochromatic gene family classification. Columns L and M: *Plasmodium* species encoding an ortholog or syntenic ortholog. Columns N and O: orthologs are heterochromatic in multiple species or a single species only. High-confidence heterochromatic genes (p > 0.99999) and potential heterochromatic genes (0.99999 > p > 0.95) are highlighted in orange and light blue, respectively. Genes with very low mappability (RPKM value <5 in at least one input sample) are highlighted in gray and were excluded from downstream analyses. This file contains six worksheets (xlsx format).

Table S2. List of All HP1-Enriched Genes that Have Orthologs in at Least One Other *Plasmodium* Species, Related to Figures 1 and 2 and Tables S1 and S7 Columns A and B: gene IDs and annotations. Column C: HP1 enrichment values (ChIP/input ratio). Column D: heterochromatic gene family classification. Columns E and F: *Plasmodium* species encoding an ortholog or syntenic ortholog. Column G: number of *Plasmodium* species in which the orthologous gene is HP1-enriched. Columns H and AB: gene IDs of orthologs in *P*. *falciparum*, *P*. *vivax*, *P*. *knowlesi*, *P*. *chabaudi*, *P*. *berghei*, and *P*. *yoelii*. HP1-enriched orthologs are highlighted in orange (xlsx format).

Table S3. Genome-wide PkHP1 Occupancy in *P*. *knowlesi* Clones A1-H.1 and A1-C.1, Related to Figure 3 and Tables S1 and S7 ChIP/input enrichment values were calculated over a 1,000-bp window (ATG ±500 bp) for each coding sequence of the *P*. *knowlesi* reference genome in *P*. *knowlesi* clones A1-H.1 and A1-C.1 schizont stages. Column A: chromosome number. Columns B and C: nucleotide positions at the start and end of each 1,000-bp window. Columns D and E: gene IDs and annotations. Columns F–I: RPKM values for the α-ChIP and input samples for each of the two clones. Columns J and K: PkHP1 enrichment values (ChIP/input ratio) for each of the two clones. Column L: fold change in PkHP1 occupancy in A1-H.1 compared with A1-C.1. Genes with ≥2.5-fold higher or lower PkHP1 occupancy in A1-H.1 are highlighted in light or dark orange, respectively. The ChIP-seq values for *P*. *knowlesi* clone A1-C.1 are identical to those listed in Table S1. Genes with very low mappability (RPKM value <5 in at least one input sample) are highlighted in gray and were excluded from downstream analyses (xlsx format).

Table S4. Genome-wide HP1 Occupancy in Four Different *P*. *falciparum* Strains, Related to Figures 4 and S4 and Tables S1 and S7 ChIP/input enrichment values were calculated over a 1,000-bp window (ATG ±500 bp) for each coding sequence of the *P*. *falciparum* 3D7 reference genome in schizont stages of *P*. *falciparum* strains Pf2004, NF135, NF54, and 3D7. Column A: chromosome number. Columns B and C: nucleotide positions at the start and end of each 1,000-bp window. Columns D and E: gene IDs and annotations. Columns F–M: RPKM values for the α-ChIP and input samples for each of the four strains. Columns N–Q: PfHP1 enrichment values (ChIP/input ratio) for each of the four strains. Columns R–U: *Z*-score-transformed ChIP enrichment values for each of the four strains. Column V: k-means cluster numbers (1–14). Column W: gene family classification. Column X: heterochromatic pseudogenes. Column Y: genes previously shown to be variantly expressed *in vitro* (Rovira-Graells et al., 2012). Column Z: degree of variation in gene expression (SD) in field samples (Mok et al., 2015). Genes with variable PfHP1 occupancy between the strains are marked in blue (k-means clusters 5–11). Genes with very low mappability (RPKM value <5 in at least one input sample) are highlighted in gray and were excluded from downstream analyses. The ChIP-seq values for *P*. *falciparum* 3D7 schizonts are identical to those listed in Tables S1 and S5 (xlsx format).

Table S5. Genome-wide PfHP1 Occupancy in Ring Stages, Trophozoites, and Schizonts, Related to Figure 5 and Tables S1 and S7 ChIP/input enrichment values were calculated over a 1000-bp window (ATG ± 500 bp) for each coding sequence of the *P*. *falciparum* 3D7 reference genome in ring stages, trophozoite, and schizont stages of *P*. *falciparum* 3D7 parasites. Column A: chromosome number. Columns B and C: nucleotide positions at the start and end of each 1,000-bp window. Columns D and E: gene IDs and annotations. Columns F–H: RPKM values for the α-ChIP samples for each of the three IDC stages. Column I: RPKM values for the input sample from schizonts. Columns J–L: PfHP1 enrichment values (ChIP/input ratio) for each of the three IDC stages. Column M: peak transcript abundance during the IDC (RNA-seq RPKM values from Kensche et al., 2016). Column N: genes previously shown to be variantly expressed in 3D7 parasites (Rovira-Graells et al., 2012). Genes with very low mappability (RPKM value <5 in the input sample) are highlighted in gray and were excluded from downstream analyses. The ChIP-seq values for *P*. *falciparum* 3D7 schizonts are identical to those listed in Tables S1 and S4 (xlsx format).

Table S6. Genome-wide HP1 Occupancy in *P. falciparum* Schizonts and Gametocytes, Related to Figures 6 and S5 and Tables S4 and S7 ChIP/input enrichment values were calculated over a 1,000-bp window (ATG ±500 bp) for each coding sequence of the *P*. *falciparum* 3D7 reference genome in schizont stages, stage II/III, and stage IV/V gametocytes of *P*. *falciparum* strain Pf2004. Column A: chromosome number. Columns B and C: nucleotide positions at the start and end of each 1,000-bp window. Columns D and E: gene IDs and annotations. Columns F–K: RPKM values for the α-ChIP and input samples for each of the three stages. Columns L–N: PfHP1 enrichment values (ChIP/input ratio) for each of the three stages. Columns O–Q: *Z*-score-transformed ChIP enrichment values for each of the three stages. Column R: k-means cluster numbers (1–12). Genes with lower or higher PfHP1 occupancy in gametocytes compared with schizonts are marked in green (k-means cluster 5) or blue (k-means clusters 6–8), respectively. Genes with very low mappability (RPKM value <5 in at least one input sample) are highlighted in gray and were excluded from downstream analyses. The ChIP-seq values for *P*. *falciparum* Pf2004 schizonts are identical to those listed in Table S4 (xlsx format).

Table S7. ChIP-Seq Libraries, Related to Tables S1–S6 and STAR Methods Column A: information on the species, strains, clones, and stages of *Plasmodium* parasite samples harvested for ChIP-seq experiments. Column B: DNA sample type used for library preparation. Column C: antibodies used in the ChIP experiment. Column D: number of PCR cycles used in the first and second library amplification. Columns E and F: Number of reads before filtering (total) and after filtering for uniquely mapping reads (unique). Column G: versions of the reference genomes used for read mapping.

Document S2. Article plus Supplemental Information
