# Supplementary material for: Comparative Heterochromatin Profiling Reveals Conserved and Unique Epigenome Signatures Linked to Adaptation and Development of Malaria Parasites
Source: Cell Host Microbe. 2018 Mar 14;23(3):407–420.e8. doi: 10.1016/j.chom.2018.01.008 (PMC5853956; doi:10.1016/j.chom.2018.01.008)
Supplement: Document S1. Figures S1–S5 [file mmc1.pdf]

Cell Host & Microbe, Volume *23*

Supplemental Information

Comparative Heterochromatin Profiling Reveals Conserved and Unique Epigenome Signatures Linked to Adaptation and Development of Malaria Parasites

Sabine A. Fraschka, Michael Filarsky, Regina Hoo, Igor Niederwieser, Xue Yan Yam, Nicolas M.B. Brancucci, Franziska Mohring, Annals T. Mushunje, Ximei Huang, Peter R. Christensen, Francois Nosten, Zbynek Bozdech, Bruce Russell, Robert W. Moon, Matthias Marti, Peter R. Preiser, Richárd Bártfai, and Till S. Voss

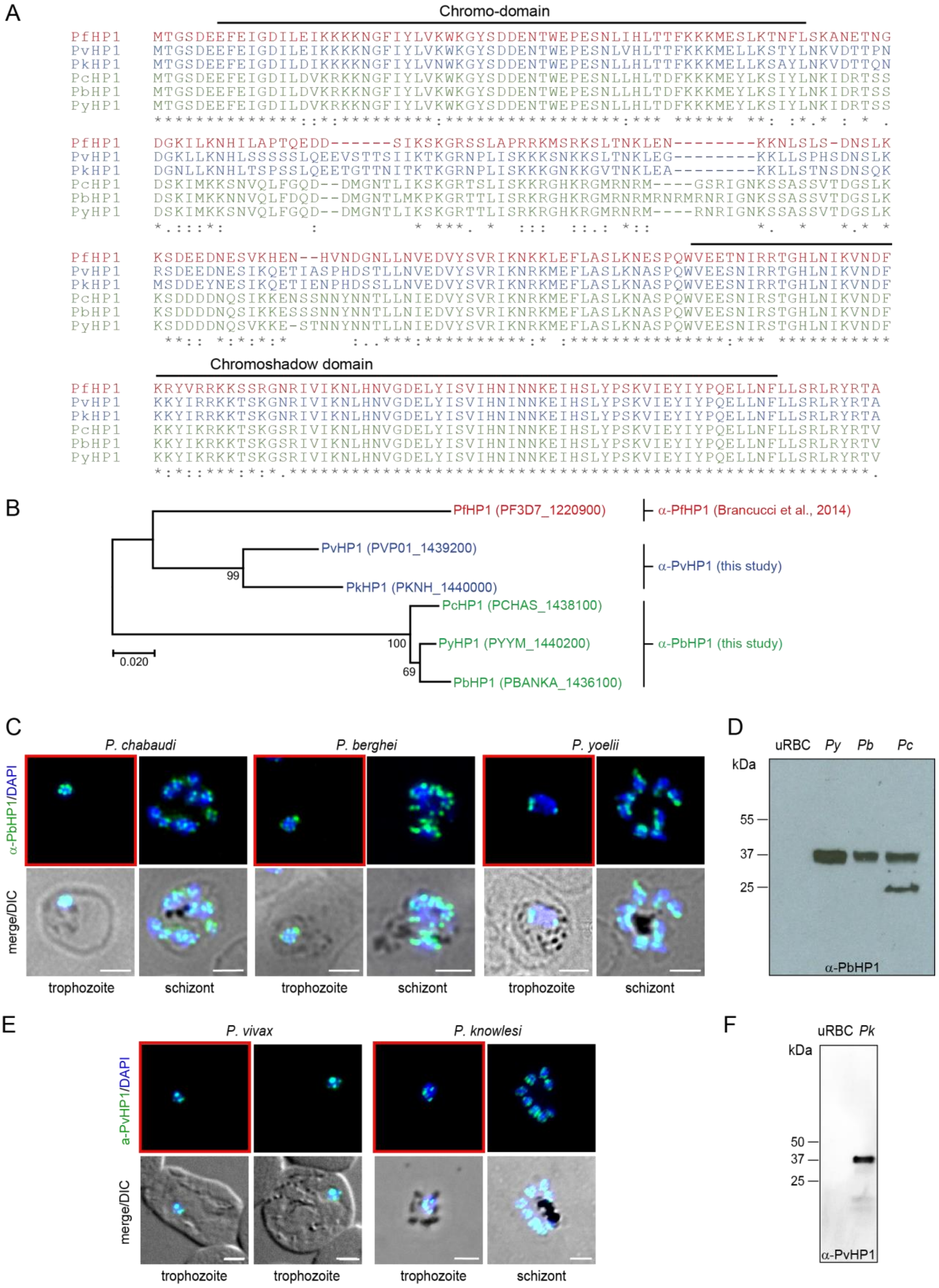

A
Chromo-domain
PfHP1 MTGSDEEFEIGDILEIKKKKNGFIYLVKWKGYSDDENTWEPESNLIHLTTFKKKMESLKTNFLSKANETNG
PvHP1 MTGSDEEFEIGDILEIKKKKNGFIYLVKWKGYSDDENTWEPESNLVHLTTFKKKMELLKSTYLNKVDTTPN
PkHP1 MTGSDEEFEIGDILDIKKKKNGFIYLVNWKGYSDDENTWEPESNLLHLTTFKKKMELLKSAYLNKVDTTQN
PcHP1 MTGSDEEFEIGDILDVKRKKNGFIYLVKWKGYSDDENTWEPESNLLHLTDFKKKMEYLKSIYLNKIDRTSS
PbHP1 MTGSDEEFEIGDILDVKRKKNGFIYLVKWKGYSDDENTWEPESNLLHLTDFKKKMEYLKSIYLNKIDRTSS
PyHP1 MTGSDEEFEIGDILDVKRKKNGFIYLVKWKGYSDDENTWEPESNLLHLTDFKKKMEYLKSIYLNKIDRTSS
PfHP1 DGKILKNHILAPTQEDD------SIKSKGRSSLAPRRKMSRKSLTNKLEN--------KKNLSLS-DNSLK
PvHP1 DGKLLKNHLSSSSSLQEEVSTTSIIKTKGRNPLISKKKSNKKSLTNKLEG--------KKLLSPHSDNSLK
PkHP1 DGNLLKNHLTSPSSLQEETGTTNITKTKGRNPLISKKKGNKKGVTNKLEA--------KKLLSTNSDNSQK
PcHP1 DSKIMKKSNVQLFGQD--DMGNTLIKSKGRTSLISKKRGHKRGMRNRM----GSRIGNKSSASSVTDGSLK
PbHP1 DSKIMKKNNVQLFDQD--DMGNTLMKPKGRTTLISRKRGHKRGMRNRMRNRMRNRIGNKSSASSVTDGSLK
PyHP1 DSKIMKKSNVQLFGQD--DMGNTLIKSKGRTTLISRKRGHKRGMRNRM----RNRIGNKSSASSVTDGSLK
PfHP1 KSDEEDNESVKHEN--HVNDGNLLNVEDVYSVRIKNKKLEFLASLKNESPQWVEETNIRRTGHLNIKVNDF
PvHP1 RSDEEDNESIKQETIASPHDSTLLNVEDVYSVRIKNRKMEFLASLKNASPQWVEESNIRRTGHLNIKVNDF
PkHP1 MSDDEYNESIKQETIENPHDSSLLNVEDVYSVRIKNRKMEFLASLKNASPQWVEESNIRRTGHLNIKVNDF
PcHP1 KSDDDDNQSIKKENSSNNYNNTLLNIEDVYSVRIKNRKMEFLASLKNASPQWVEESNIRSTGHLNIKVNDF
PbHP1 KSDDDDNQSIKKESSSNNYNNTLLNIEDVYSVRIKNRKMEFLASLKNASPQWVEESNIRSTGHLNIKVNDF
PyHP1 KSDDDDNQSVKKE-STNNYNNTLLNIEDVYSVRIKNRKMEFLASLKNASPQWVEESNIRSTGHLNIKVNDF
Chromoshadow domain
PfHP1 KRYVRRKKSSRGNRIVIKNLHNVGDELYISVIHNINNKEIHSLYPSKVIEYIYPQELLNFLLSRLRYRTA
PvHP1 KKYIRRKKTSKGNRIVIKNLHNVGDELYISVIHNINNKEIHSLYPSKVIEYIYPQELLNFLLSRLRYRTA
PkHP1 KKYIRRKKTSKGNRIVIKNLHNVGDELYISVIHNINNKEIHSLYPSKVIEYIYPQELLNFLLSRLRYRTA
PcHP1 KKYIKRKKTSKGSRIVIKNLHNVGDELYISVIHNINNKEIHSLYPSKVIEYIYPQELLNFLLSRLRYRTV
PbHP1 KKYIKRKKTSKGSRIVIKNLHNVGDELYISVIHNINNKEIHSLYPSKVIEYIYPQELLNFLLSRLRYRTV
PyHP1 KKYIKRKKTSKGSRIVIKNLHNVGDELYISVIHNINNKEIHSLYPSKVIEYIYPQELLNFLLSRLRYRTV
B
PfHP1 (PF3D7_1220900)
α-PfHP1 (Brancucci et al., 2014)
PvHP1 (PVP01_1439200)
99
PkHP1 (PKNH_1440000)
α-PvHP1 (this study)
PcHP1 (PCHAS_1438100)
100
PyHP1 (PYYM_1440200)
69
PbHP1 (PBANKA_1436100)
α-PbHP1 (this study)
0.020
C
P. chabaudi
P. berghei
P. yoelii
α-PbHP1/DAPI
merge/DIC
trophozoite
schizont
trophozoite
schizont
trophozoite
schizont
D
uRBC
Py
Pb
Pc
kDa
55
37
25
α-PbHP1
E
P. vivax
P. knowlesi
a-PvHP1/DAPI
merge/DIC
trophozoite
trophozoite
trophozoite
schizont
F
uRBC
Pk
kDa
50
37
25
α-PvHP1

Figure S1. HP1 orthologs in six *Plasmodium* species (related to Figure 1 and STAR Methods).

(A) Multiple sequence alignment of *Plasmodium* HP1 orthologs. Conserved residues are marked by asterisks. The positions of the PfHP1 chromo- and chromoshadow domains (Flueck et al., 2009) are indicated above the alignment.

(B) Phylogenetic tree of *Plasmodium* HP1 orthologs. Bootstrap values are indicated at the nodes (based on 1'000 replicates). Antibodies used to immune-precipitate HP1-associated chromatin from the six *Plasmodium* species for ChIP-seq experiments are shown on the right.

(C) Representative IFA images obtained with α-PbHP1 antibodies showing perinuclear HP1 localisation (green) in *P. chabaudi*, *P. berghei* and *P. yoelii* trophozoite and schizont stage parasites. Nuclei were stained with DAPI (blue). DIC, differential interference contrast. Scale bar, 2.5 μm. The red-framed images are identical to those presented in Figure 1A.

(D) α-PbHP1 Western blot analysis detects a single protein at sizes expected for PyHP1, PbHP1 and PcHP1 (~32-33 kDa) in whole cell lysates generated from *P. yoelii* (lane 2), *P. berghei* (lane 3) and *P. chabaudi* (lane4), respectively, but not in uninfected RBCs (lane 1; negative control). The lower band in lane 4 may represent a proteolytic degradation product of PcHP1. Generation of the polyclonal affinity-purified α-PbHP1 antibodies is explained in detail in the STAR Methods.

(E) Representative IFA images obtained with α-PvHP1 antibodies showing perinuclear HP1 localisation (green) in *P. vivax* trophozoites (note that schizont stages were not observed in this sample) and *P. knowlesi* trophozoite and schizont stage parasites. Nuclei were stained with DAPI (blue). DIC, differential interference contrast. Scale bar, 2.5 μm. The red-framed images are identical to those presented in Figure 1A.

(F) α-PvHP1 Western blot analysis detects a single protein at the expected size for PkHP1 (~32 kDa) in whole cell lysates generated from *P. knowlesi* (lane 2), but not in uninfected RBCs (lane 1; negative control). Generation of the polyclonal affinity-purified α-PvHP1 antibodies is explained in detail in the STAR Methods.

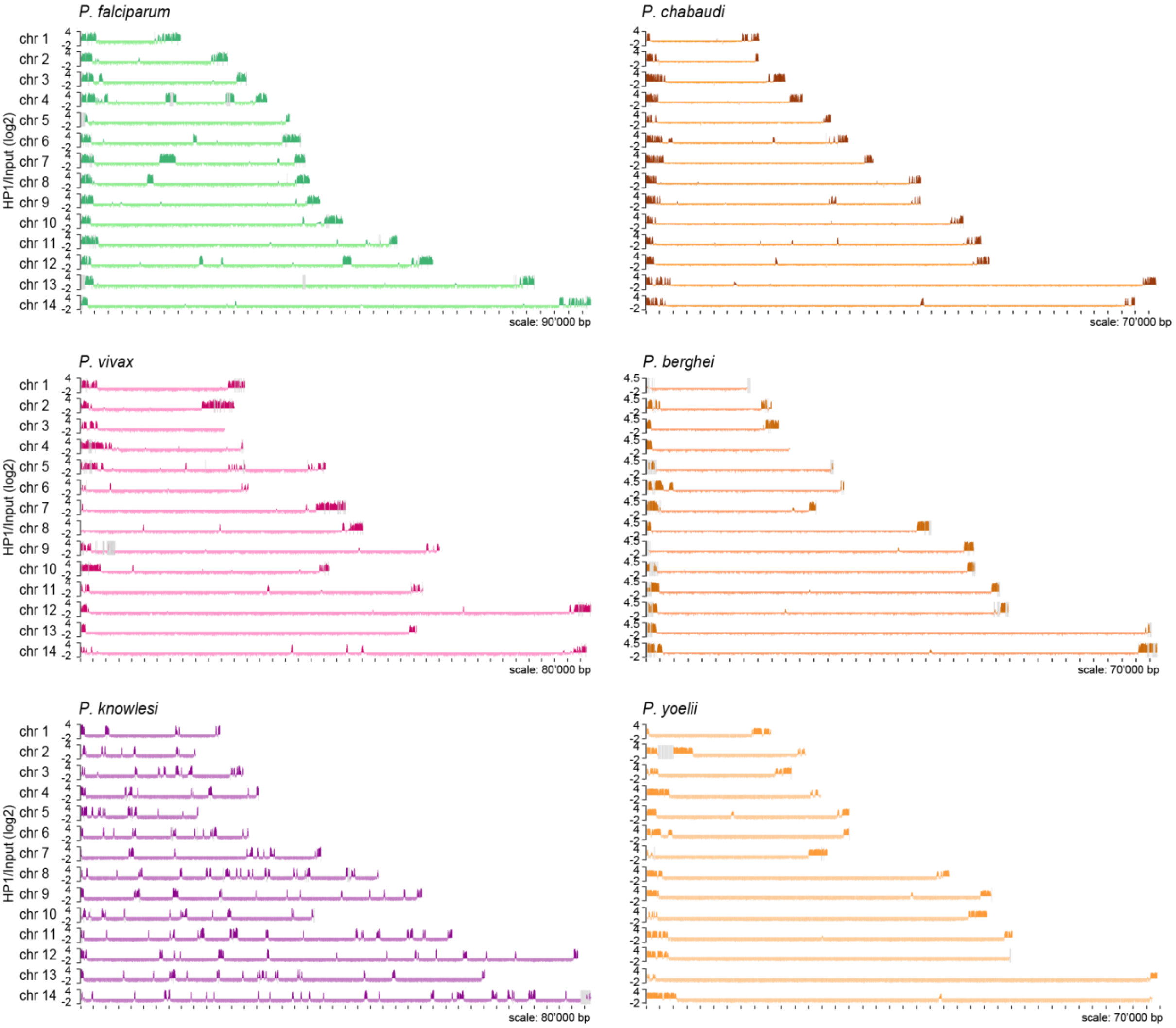

Figure S2: Genome-wide overviews of HP1 localization in schizont stages of six different *Plasmodium* species (related to Figures 1 and S3).

Chromosome maps depicting HP1-demaracted heterochromatin over all 14 chromosomes of schizont stages from *P. falciparum* (3D7), *P. vivax*, *P. knowlesi* (A1-C.1), *P. chabaudi*, *P. berghei* and *P. yoelii*. Log2-transformed HP1-ChIP/Input ratios were calculated in 1000 bp windows. Regions with low mappability are highlighted by grey bars.

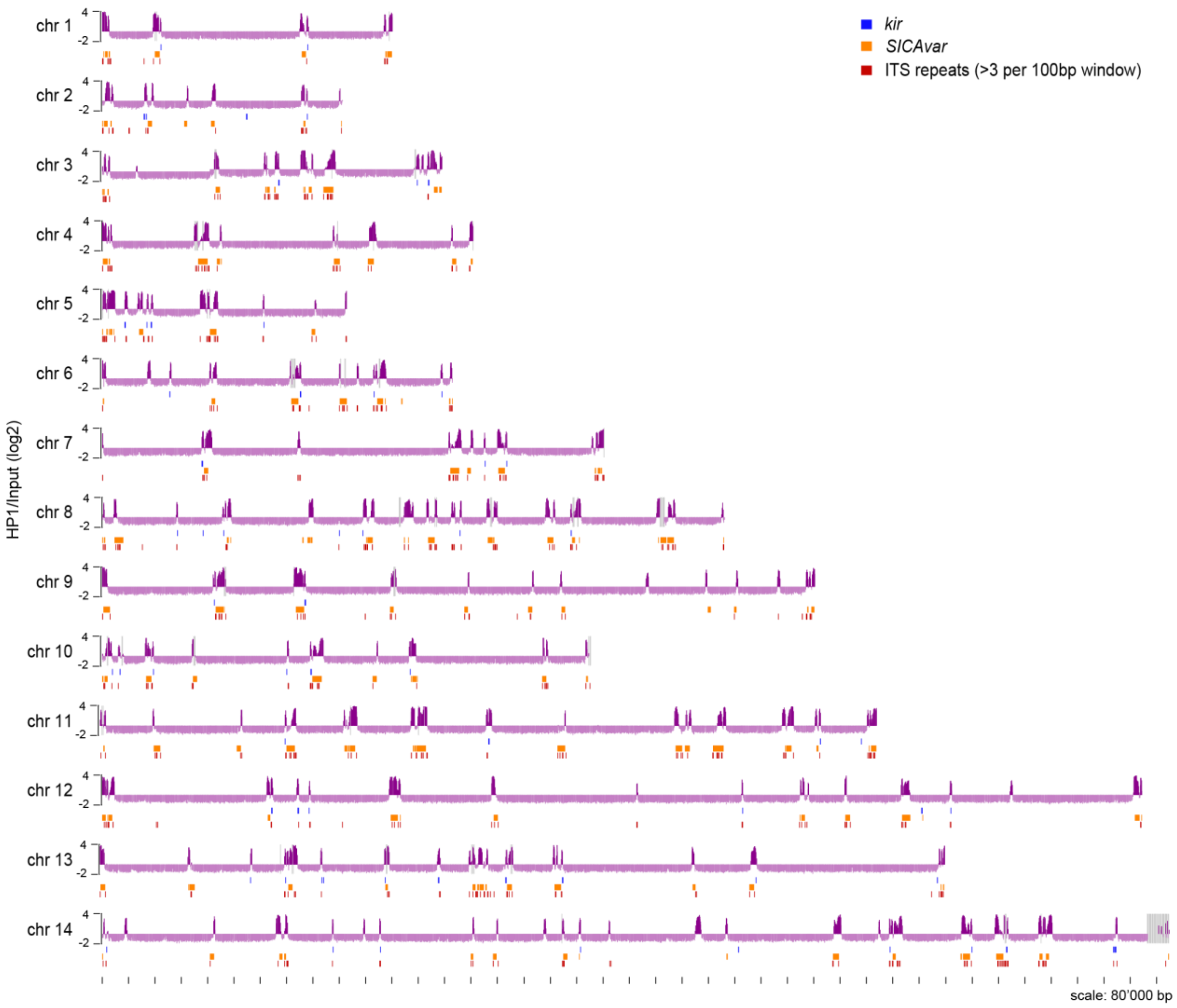

Figure S3: Genome-wide association of *SICAvar* genes, *kir* genes and ITS elements with heterochromatic regions in *P. knowlesi* (related to Figure 1).

Chromosome maps depicting HP1-demaracted heterochromatin on all 14 chromosomes in *P. knowlesi* (A1-C.1). Log2-transformed HP1-ChIP/Input ratios were calculated in 1000 bp windows. Coding sequences of *kir*/*kir*-like genes and *SICAvar* genes are depicted in blue and orange, respectively. 100 bp windows containing three or more interstitial telomere repeat sequences (ITSs) are marked in red. Regions with low mappability are highlighted by grey bars.

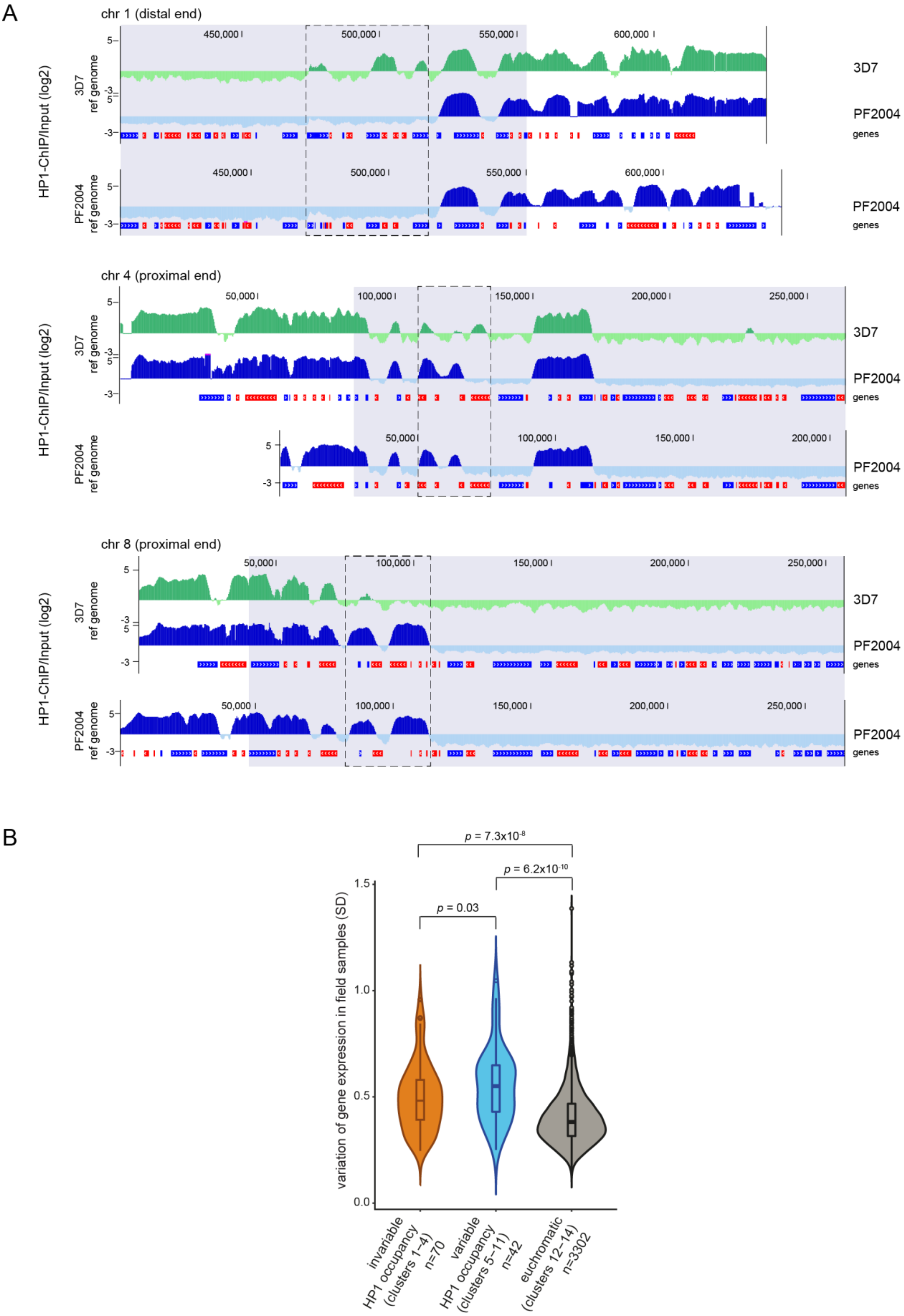
A
chr 1 (distal end)
450,000
500,000
550,000
600,000
HP1-ChIP/Input (log2)
3D7 ref genome
PF2004 ref genome
5
-3
3D7
PF2004
genes
chr 4 (proximal end)
50,000
100,000
150,000
200,000
250,000
chr 8 (proximal end)
B
p = 7.3x10-8
p = 6.2x10-10
p = 0.03
variation of gene expression in field samples (SD)
1.5
1.0
0.5
0.0
invariable HP1 occupancy (clusters 1–4) n=70
variable HP1 occupancy (clusters 5–11) n=42
euchromatic (clusters 12–14) n=3302

Figure S4. Strain-specific differences in heterochromatin organization in syntenic regions of the genome (related to Figure 4 and Table S4).

(A) Log2-transformed PfHP1-ChIP/Input ratio tracks generated from *P. falciparum* Pf2004 and 3D7 schizont stage parasites. The upper tracks were generated by mapping 3D7 (green) and Pf2004 (blue) ChIP-seq reads against the 3D7 reference genome (PlasmoDB v26). The Pf2004 ChIP-seq data were also mapped against the matching *P. falciparum* Pf2004 genome (lower tracks). Syntenic regions are marked in grey and regions with changing PfHP1 occupancy within these regions are framed. Coding sequences are shown as blue (sense strand) and red (antisense strand) boxes.

(B) Violin plot of gene expression variation in the heterochromatic and euchromatic groups of genes. Y-axis: standard deviation (SD) of relative gene expression levels determined by microarray analysis in 549 *P. falciparum* field samples (Mok et al., 2015) (only group A isolates, which all consisted of young ring stage parasites and displayed consistently low gametocyte densities (Mok et al., 2015), were included in this analysis). X-axis: genes with invariable HP1 occupancy (clusters 1-4) and variable HP1 occupancy (clusters 5-11) are shown in brown and blue, respectively. Euchromatic genes (clusters 12-14) are shown in grey. *p* values of Wilcoxon Rank Sum tests are shown on top for each pair of groups.

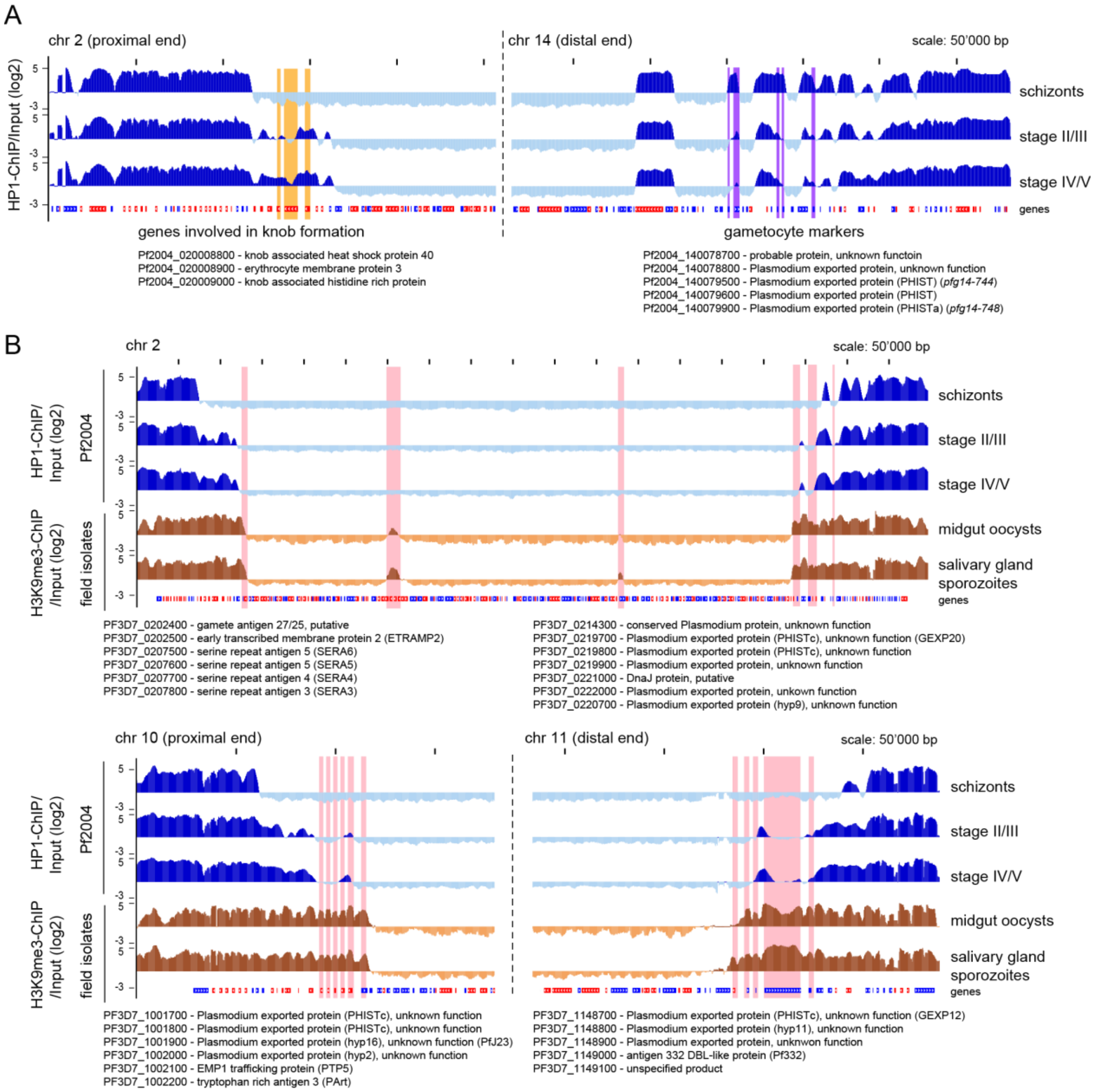

Figure S5. Altered heterochromatin organization in *P. falciparum* gametocytes, midgut oocysts and salivary gland sporozoites (related to Figure 6 and Table S6).

(A) Log2-transformed PfHP1-ChIP/Input ratio tracks generated from *P. falciparum* Pf2004 schizonts and stage II/III and IV/V gametocytes. ChIP-seq reads were aligned against the Pf2004 genome and depicted as in Figure 6A. Genes involved in knob formation (orange) and early gametocyte markers (purple) are highlighted and their Pf2004 Gene IDs are shown. Coding sequences are shown as blue (sense strand) and red (antisense strand) boxes.

(B) Log2-transformed PfHP1-ChIP/Input ratio tracks from different *P. falciparum* life cycle stages mapped against the 3D7 reference genome. The heterochromatic domains on chromosome 2 and zoom-in views of the proximal and distal ends of chromosome 10 and 11, respectively, are depicted as representative examples. PfHP1-ChIP/Input ratio tracks of Pf2004 schizonts, stage II/III and IV/V gametocytes are depicted in blue. H3K9me3-ChIP/Input ratio tracks of midgut oocysts and salivary gland sporozoites from *P. falciparum* field isolates (Gomez-Diaz et al., 2017) are depicted in brown. Genes differentially marked in sporozoites compared to gametocytes are highlighted in pink (the corresponding Gene IDs are listed below the tracks).
